# Kidney stone growth through the lens of Raman mapping

**DOI:** 10.1038/s41598-024-61652-9

**Published:** 2024-05-12

**Authors:** John W. Robinson, William W. Roberts, Adam J. Matzger

**Affiliations:** 1 Department of Chemistry, University of Michigan, Ann Arbor, MI 48109 USA; 2 Division of Endourology, Department of Urology, University of Michigan, Ann Arbor, MI 48109 USA; 3 Department of Biomedical Engineering, University of Michigan, Ann Arbor, MI 48109 USA; 4 Macromolecular Science and Engineering Program, University of Michigan, Ann Arbor, MI 48109 USA

**Keywords:** Hyperspectral imaging, Vibrational spectroscopy, Urology, Biomineralization, Biomineralization, Renal calculi, Imaging studies

## Abstract

Bulk composition of kidney stones, often analyzed with infrared spectroscopy, plays an essential role in determining the course of treatment for kidney stone disease. Though bulk analysis of kidney stones can hint at the general causes of stone formation, it is necessary to understand kidney stone microstructure to further advance potential treatments that rely on in vivo dissolution of stones rather than surgery. The utility of Raman microscopy is demonstrated for the purpose of studying kidney stone microstructure with chemical maps at ≤ 1 µm scales collected for calcium oxalate, calcium phosphate, uric acid, and struvite stones. Observed microstructures are discussed with respect to kidney stone growth and dissolution with emphasis placed on < 5 µm features that would be difficult to identify using alternative techniques including micro computed tomography. These features include thin concentric rings of calcium oxalate monohydrate within uric acid stones and increased frequency of calcium oxalate crystals within regions of elongated crystal growth in a brushite stone. We relate these observations to potential concerns of clinical significance including dissolution of uric acid by raising urine pH and the higher rates of brushite stone recurrence compared to other non-infectious kidney stones.

## Introduction

Chemical mapping using Raman microscopy has long been used to better understand the growth and function of biomineral systems including bones^1,2^, teeth^3,4^, and, more recently, kidney stones (urinary calculi)^5–8^. Compositional and/or structural maps of urinary calculi have been produced using micro computed tomography (µCT)^6–15^, fluorescence microscopies^6–8^, electron microscopies^6,7,16,17^, optical microscopies^6–8,18^, multi-photon spectroscopy^19^, infrared microscopy^20–22^, and Raman microscopy^5–8^. Micro computed tomography allows complete volume imaging of intact stones but has limited voxel resolution of 2–5 µm depending on stone size^14^. Fluorescence microscopies allow imaging on similar length scales compared to Raman microscopy^6–8^; however, autofluorescence in kidney stones is primarily sensitive to organic components whereas Raman spectroscopy provides direct information about mineral composition^6–8^. Electron microscopy has much higher resolution (~ 1 nm) than other techniques but requires significant sample preparation and high vacuum that can dehydrate certain minerals over time. Optical microscopies, including circular and crossed polarized light^6–8,18^, require stone destruction in the form of thin sectioning. Multiphoton spectroscopy provides mineral phase identification but requires specialized laser systems^19^. Reflectance-mode infrared microscopy offers similar vibrational information as Raman spectroscopy but spatial resolution is limited to > 5 µm by the diffraction limit of infrared light^22^ or the size of attenuated total reflectance probes^23^. In contrast, commercially available Raman microscopes can map with spatial resolution < 1 µm while providing direct identification of minerals through their vibrational signatures.

Vibrational spectroscopy has a long history of use for analysis of urinary stones^24–26^ and infrared spectroscopy of ground stone samples is commonly used to identify bulk components for clinical purposes^25^. In contrast, use of Raman spectroscopy for compositional analysis of urinary stones has largely been confined to use in research settings^24,27–29^. Early work by Daudon identified the potential utility of Raman microprobe analysis for identification of small features involved in nucleation of urinary stones^24^. A recent review by Lucas, Bazin, and Daudon noted opportunities for using Raman spectroscopy to study various pathological biomineralizations and highlighted the mapping of kidney stones at 5 µm spatial resolution^5,21,28^. As we demonstrate in this work, achieving 0.5–1 µm scale Raman mapping allows insight into kidney stone growth that would be missed by µCT.

Raman mapping of biological samples is often difficult due to autofluorescence caused by biomolecules and heating damage caused by high-powered lasers. Biomolecule autofluorescence may be avoided by use of longer wavelengths for excitation but with a concomitant reduction in Raman intensity proportional to λ^-4^ and a degradation of achievable spatial resolution. With Raman scattering, longer wavelength lasers often necessitate longer exposure times and/or higher laser power to achieve the same signal/noise as shorter wavelength lasers. In kidney stones, this increased exposure duration increases the risk of sample damage by mineral decomposition or carbonization of intercrystalline biomolecules.

A common technique to reduce autofluorescence is to rely on photobleaching induced by the lasers used for Raman scattering with autofluorescence reduced by a factor of 80% in bone samples using a 532 nm laser^30^. Unlike the apatite found in bone, certain minerals found in kidney stones, like brushite (CaHPO_4_·2H_2_O) and struvite (MgNH_4_PO_4_·6H_2_O), will dehydrate under intense radiation and cannot be laser photobleached without risking sample damage. As an alternative to photobleaching, a hydrogen peroxide + light treatment has recently been shown to reduce autofluorescence in biological samples^31^. This approach has been adapted here to improve signal to noise in Raman spectra for kidney stones allowing mapping of majority brushite and struvite stones with a 532 nm laser. The improved sensitivity has allowed us to observe calcium oxalate and apatite crystals with ~ 1 µm dimensions within brushite stones and struvite stones for the first time.

Here we report results of Raman mapping for six samples taken from five different kidney stones. The six most common minerals found in kidney stones are represented: calcium oxalate monohydrate (COM), calcium oxalate dihydrate (COD), hydroxyapatite (HAp), uric acid (UA), brushite, and struvite. Each sample is mapped at 1 µm or 0.5 µm spatial resolution, an order of magnitude improvement over previous studies using Raman mapping^5^. This enables observation of micrometer-sized features that appear related to changes in kidney stone growth. The crystal orientation of COM is correlated to changes in Raman peak intensity allowing basic orientational mapping of COM when using polarized lasers for Raman excitation. The origin of minor constituents is discussed in relation to crystallization conditions including < 10 µm wide rings found in urinary calculi that are otherwise formed of steady growth of a single mineral. We present an example of depth profiling in a struvite stone and discuss its potential application in the study of laser lithotripsy.

## Results and discussion

**Figure 1:**
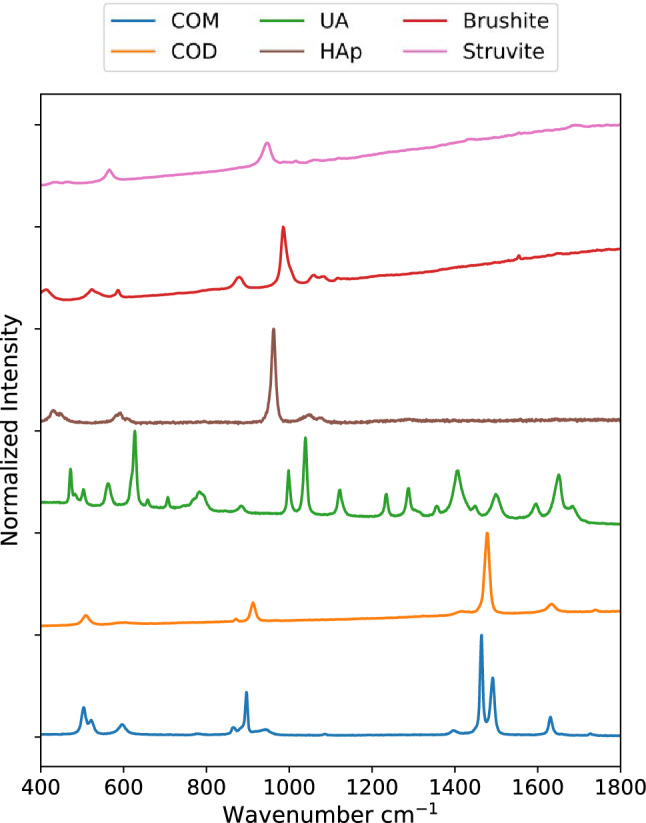
Raman spectra for common kidney stone minerals. Raman spectra were collected using powders of calcium oxalate monohydrate, calcium oxalate dihydrate, uric acid, hydroxyapatite, brushite, and struvite minerals from 400 to 1800 cm^−1^**.** The uric acid spectrum was collected using a 785 nm laser. All others were collected using a 532 nm laser.

Raman spectra for samples of COM, COD, HAp, UA, brushite, and struvite powders are shown in Fig. 1. These were used subsequently for least-squares fitting of mapping data. Prominent peaks useful for mineral identification are listed in Table 1 along with vibrational mode assignments, if available. Separate spectra for each mineral are included in the Supporting Information (Figs. S1–S7). Brushite and struvite both weakly fluoresce when irradiated with 532 nm light which results in the rising background seen in Fig. 1.

**Table 1:** Prominent Raman peaks of kidney stone minerals in the 500–1600 cm^−1^ range.

Mineral	Peak position	Relative peak intensity	Notes
COM	1491	0.55	Carboxylate symmetric stretch^32^
			Intensity varies with crystal orientation
	1464	1	Carboxylate symmetric stretch^32^
			Intensity varies with crystal orientation
	897	0.35	Carboxylate bend^32^
COD	1478	1	Carboxylate stretch^32^
	913	0.22	Carboxylate bend^32^
Uric acid	626	1	
	999	0.56	
	1039	1	
	1407	0.65	
Hydroxyapatite	962	1	Phosphate ν_1_ stretch^33^
			Shifts to 958 cm^−1^ in carbonate apatite
	611	0.026	Phosphate ν_4_ stretch^33^
			Overlaps with peak at 589 cm^−1^
	589	0.099	Overlaps with peak at 611 cm^-1^
			Double/shouldered peak near 600 cm^−1^ distinguishes carbonate apatite from struvite
Brushite	985	1	Phosphate ν_2_ stretch^34^
	993	0.45	Appears as shoulder to more intense phosphate stretch at 985 cm^-1^
	878	0.26	Phosphate ν_3_ stretch ^34^
Struvite	945	1	Phosphate ν_1_ stretching mode^35^
	565	0.46	Phosphate ν_4_ stretching mode
	1691	0.13	Ammonium stretching band(s)^35^

The Renishaw WiRE software was used to determine peak positions and intensities using a mixed Gaussian–Lorentzian profile curve fit. Peaks were identified in the Raman spectra from Fig. 1 after baseline correction.

### Calcium oxalate monohydrate stone

**Figure 2:**
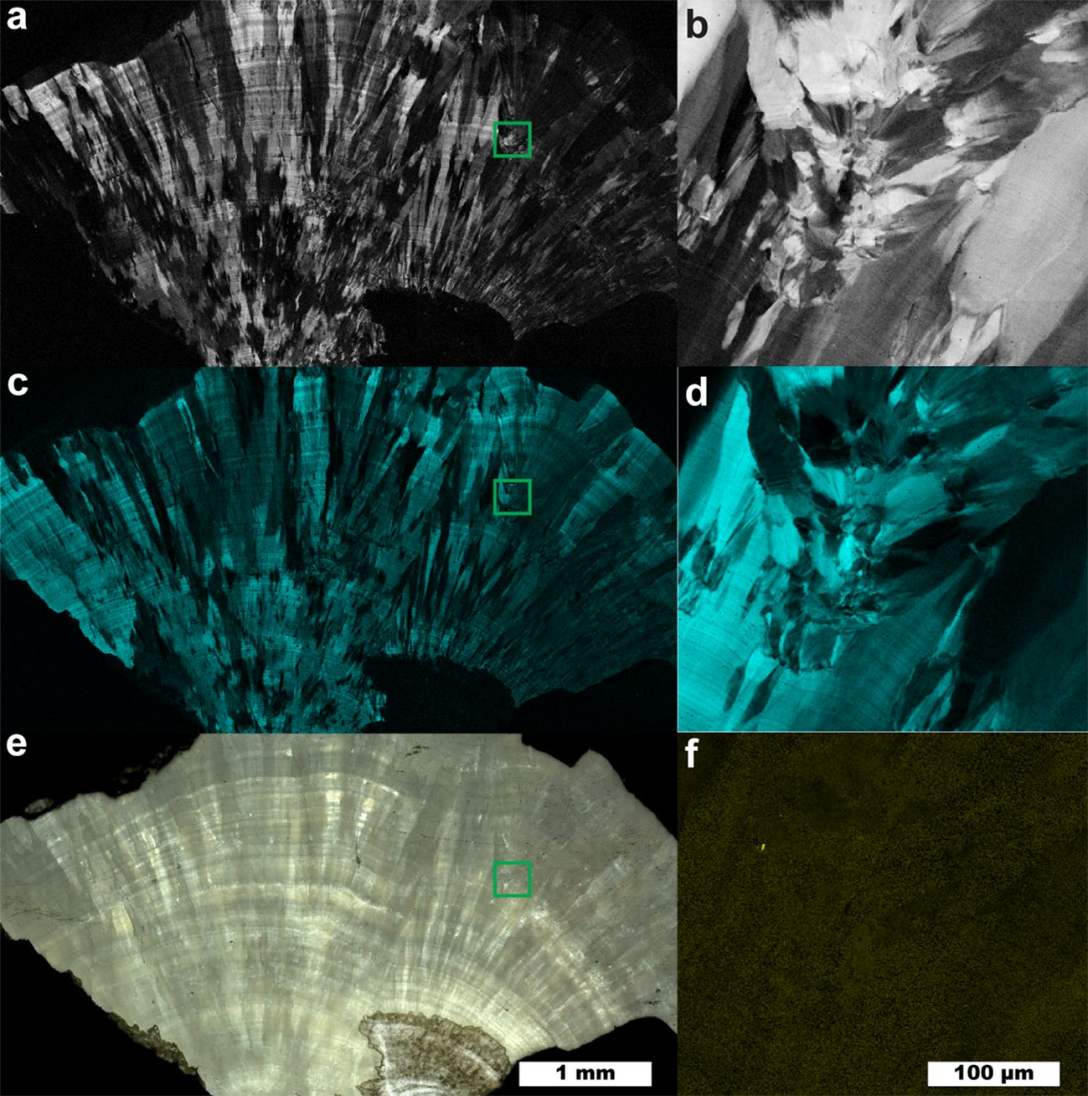
Raman map of a COM stone with orientation differences. (**a**) 5 µm spatial resolution map of COM with 1464 cm^−1^ carboxylate stretch dominant. (**b**) 1 µm spatial resolution map of inset in (**a**). (**c**) 5 µm spatial resolution map of COM with 1491 cm^−1^ carboxylate stretch dominant. (**d**) 1 µm spatial resolution map of inset in (**c**). (**e**) White light image. (**f**) 1 µm spatial resolution map of COD, inset from (**e**).

The two most prominent peaks in COM (Fig. 1) at 1464 and 1491 cm^−1^ originate from the stretching of carboxylate groups in the oxalate ion of COM^32^. The relative intensity of each depends on the orientation of COM with respect to laser polarization. In certain orientations, only one of the two carboxylate stretches may be obvious in the collected Raman spectrum (Figure S8). COM spectra with either the 1464 or 1491 cm^−1^ dominating appear qualitatively similar to the Raman spectrum of COD with its single carboxylate peak at 1476 cm^-1^. This similarity is close enough that least squares methods can erroneously identify COD as a major presence when powdered COM and COD are used for fitting (Figure S9). Similar misidentification seems to appear in the literature in Supplementary Fig. 6 of Sivaguru^6^ in which we would identify all three presented spectra as COM based on the positions of oxalate stretching peaks from 1460 to 1495 cm^−1^ and oxalate bending peaks at 890–920 cm^−1^^6^. For this reason, we recommend COM be identified in maps using at least two different spectra: one with the 1464 cm^−1^ peak dominant and a second with the 1491 cm^−1^ peak dominant. At ≈2 cm^−1^ wavenumber resolution, this is sufficient to consistently distinguish COM and COD and allows contrast of regions with differing COM orientation as shown in Fig. 2.

Figure 2 shows a large fragment from a COM stone that exhibits layered radial growth in the manner of type 1a stones according the classification of Daudon^11,17^. Using the diagenetic classifications outlined in Sivaguru et al. this fragment primarily exhibits “cortex COM” (COM_c_): COM with microscale and nanoscale layering due to embedding of biomolecules during growth^6,7^. COM_c_ typically forms during later stone growth and coats other minerals (COM, COD, or apatite) that form the nidus of growth. COM_c_ layering is exhibited over the entire region of the fragment in Fig. 2 indicating this fragment lacks the original center of the stone.

At several points there are changes in the radius of curvature in the growth layers indicating disruption in evenly layered growth. Figures 2b, c, d and f focus on one such region revealing a lack of ≤ 1 µm layering that is characteristic of COM_c_ and the presence of a ~ 3 µm COD crystal (Fig. 2f). This indicates COD nucleated or agglomerated near this site, potentially alongside COM. The lack of clear crystal facets prevents identification of the original phases present. COM_c_ layers show continued growth around the disruptive COM/COD agglomerations, eventually coating them entirely.

### Uric acid stone

**Figure 3:**
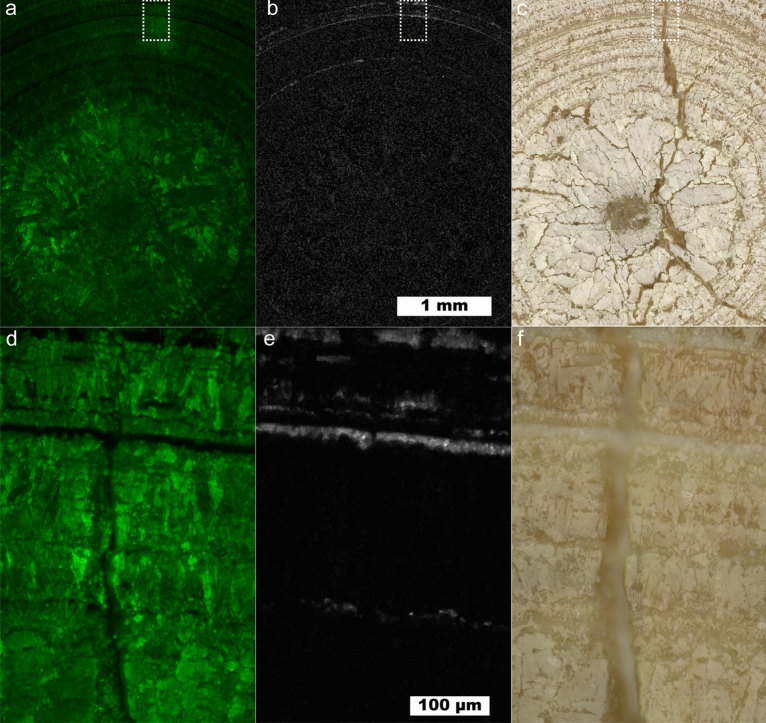
Uric acid kidney stone containing layers of COM. (**a**) Map showing intensity of uric acid at 10 µm spatial resolution. (**b**) Map of COM at 10 µm spatial resolution using the 1464 cm^−1^ peak. (**c**) White light image for (**a**) and (**b**). (**d**) 1 µm spatial resolution map of inset in (**a**). (**e**) 1 µm spatial resolution map of inset in (**b**). (**f**) White light image for (**d**) and (**e**).

Figure 3 shows a map of a predominantly uric acid stone at 10 µm and 1 µm spatial resolutions showing a cross section of an entire UA stone and the fine structure near a fracture, respectively. The interior of the uric acid stone shows larger UA crystals with numerous cracks or voids present. A lack of sharp uric acid crystal facets and the prominent voids near the center of the stone indicates the kidney stone nidus may have been composed of a less-dense mineral than uric acid. Dissolution of kinetically favored uric acid dihydrate followed by reprecipitation as uric acid is consistent with the observed porosity at the center of the stone. Uric acid dihydrate begins to convert to anhydrous uric acid in < 24 h both in air and in water^36–38^. As these samples have been stored in air for > 4 years, uric acid dihydrate is expected to have decomposed leaving poorly attached anhydrous uric acid that would have been removed during sample preparation.

A layered uric acid structure predominates in the outer regions of the stone. The radially concentric growth around the entire circumference of the stone indicates consistent exposure to urine. In the layered zone several ≤ 10 µm thick layers of COM are present. These COM layers maintain the concentric growth pattern of the uric acid suggesting COM is also deposited during consistent exposure to urine much like the COM_c_ observed in type 1a stones^6,7,11,17^. COM may have nucleated directly on the UA stone surface in an example of crystal pseudoseeding as there are known epitaxial relationships between UA and other kidney stone minerals^39^.

While > 5 µm layers of COM within uric acid can be observed by µCT, the presence of thinner, broken layers like the innermost ring of COM seen in Fig. 3e may be difficult to detect. Identifying the presence of 1 µm thick layers is important in the understanding of uric acid stone growth as even thin layers of COM not observable by µCT could act as protective barriers against uric acid dissolution and excretion. Urine pH plays a pivotal role in uric acid solubility with an exponential increase at approximately pH 5.5^40^. In contrast, COM solubility is low and relatively stable in saline solution at physiological pH^41,42^. Thus, even if urine pH is not consistently low, uric acid stones could persist in vivo due to the protective barrier provided by COM across a wide range of physiological pH.

The ability of thin COM layers to prevent uric acid dissolution will depend on a number of additional factors including their mechanical stability, completeness of coverage, and dissolution/mixing efficiency at coverage gaps. There are reports of partially successful and failed applications of dissolution therapy to mixed-phase uric acid stones containing calcium minerals^43,44^. In-vitro dissolution experiments with carefully selected human stone samples or appropriate artificial models will be necessary to understand the effect, if any, of thin COM layers on uric acid dissolution.

### Brushite stone

**Figure 4:**
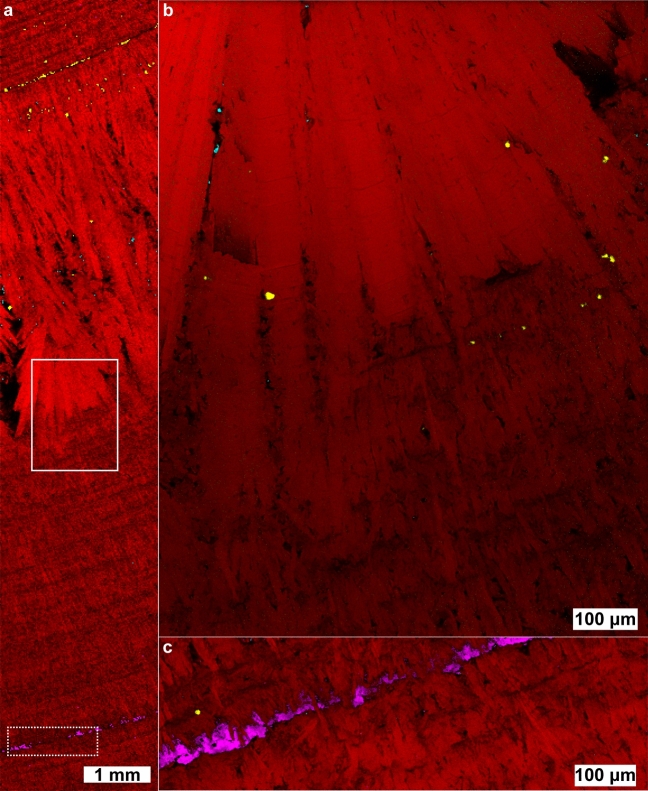
Raman chemical image of a predominantly brushite kidney stone. The stone contains brushite (red), COM (cyan), COD (yellow), and apatite (magenta). (**a**) 10 µm spatial resolution map along the length of a brushite stone. (**b**) 1 µm spatial resolution map of solid inset. (**c**) 1 µm spatial resolution map of hydroxyapatite band (dashed inset). White light images are shown in Figure S10.

The brushite stone pictured in Fig. 4 shows two distinct brushite stone morphologies: a “classic” brushite layer^15^ of elongated radial crystals between two regions of more compact brushite that resembles type 1a stones^12^. The even, layered growth in the compact brushite regions indicate continuous exposure to urine much like the layers of COM_c_ in type 1a stones. In the compact regions, there is one concentric layer of COD and one concentric layer of HAp. Otherwise, the compact regions are formed almost entirely from brushite. HAp is typically associated with higher pH solutions than brushite with HAp having a lower solubility above pH 4.1^46,47^ Despite its thermodynamic stability, other forms of calcium phosphate are typically favored kinetically, including brushite^48^. This suggests the HAp layer observed resulted from a period in which the outer brushite layer was exposed to solution allowing dissolution and reprecipitation as HAp.

The COD band in Fig. 4a occurs at the start of non-compact crystallization of brushite. Unlike more compact regions, scattered COM and COD crystals are apparent in the layer of radially elongated brushite crystals. The apparent co-precipitation of COM, COD, and brushite indicates urine was supersaturated with respect to all three minerals during growth of elongated brushite. As calcium is the common ion among the three minerals, the elongated, radial bundles of brushite may be an indicator of high calcium availability in urine which has been observed in brushite stone forming patients^49^. COD is also more commonly associated with higher calcium concentrations than COM^50^ giving additional evidence for a mechanism of increased calcium concentration rather than a simultaneous increase in both oxalate and phosphate at the onset of elongated brushite formation. The higher relative area occupied by COD crystals at the onset of elongated crystal growth indicates a higher ratio of COD/brushite growth rate. The decrease in relative calcium oxalate/brushite area toward the outer regions of elongated growth suggests the supersaturation ratio $\frac{{S}_{CaOx}}{{S}_{Brushite}}$ decreased over time leading to a lower ratio of CaOx/brushite growth rates. Oxalate is the limiting ion in calcium oxalate precipitation in typical urine with a concentration an order of magnitude lower than calcium^51^. In contrast, phosphate concentration is approximately an order of magnitude higher than calcium in typical urine which likely explains the large, continuous growth of radial brushite bundles with isolated crystals of calcium oxalate forming as additional oxalate is excreted by the kidneys.

It has been noted that faster-growing stones are more likely to reoccur in patients and brushite stones reoccur more often than other non-infectious stones^52^. However, no morphological distinction has been made between radially bundled brushite and compact brushite with both being identified as type 4d^11,52^. Given the potential for mechanistic differences between compact brushite and radially bundled brushite, we believe it would be beneficial to subdivide the classification of brushite stones by morphology for future research into recurrence.

### Mixed calcium oxalates and hydroxyapatite stone

**Figure 5:**
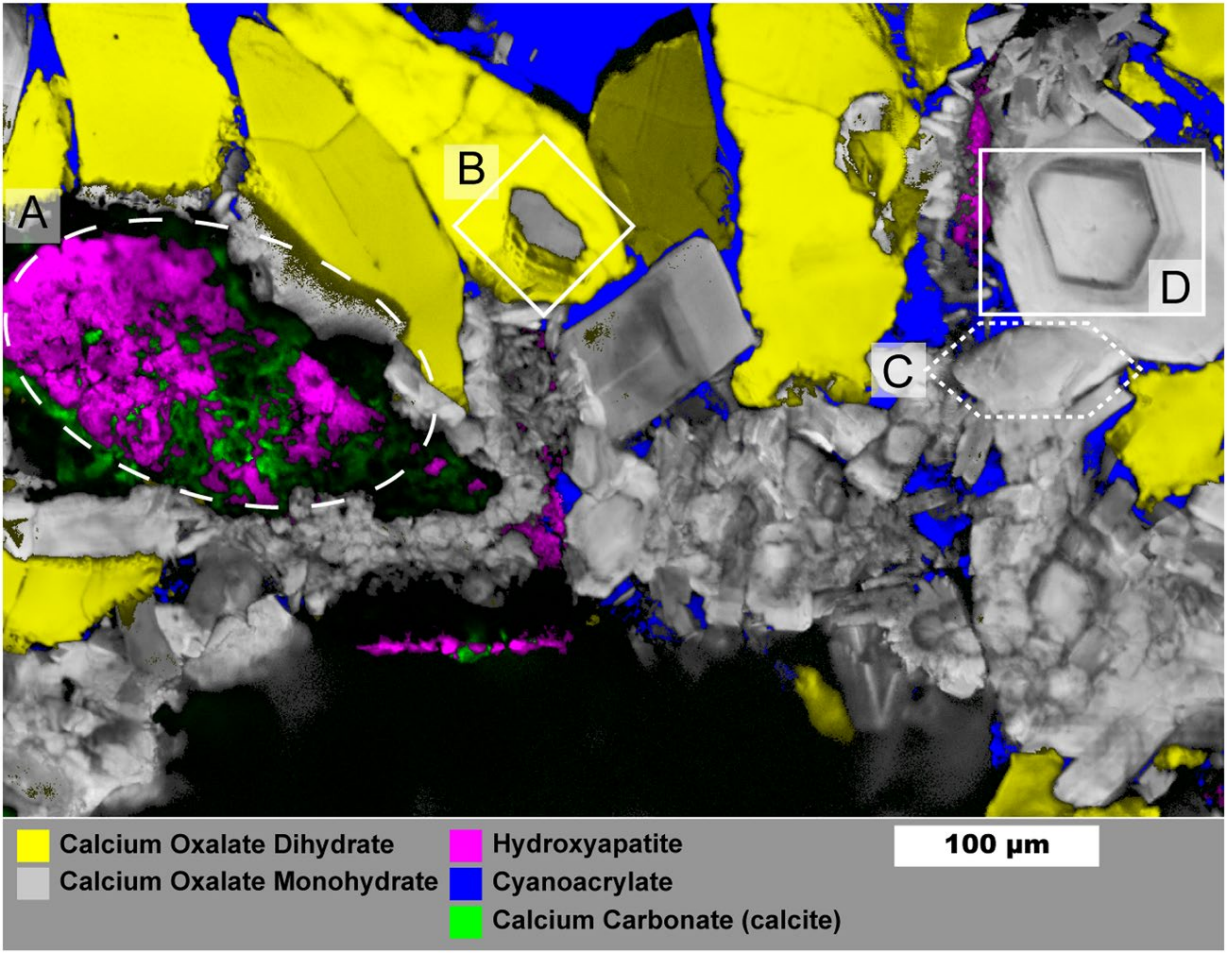
Raman image of mixed calcium oxalate and apatite stone fragment. Mapping was performed with 1 µm spatial resolution. Region (**A**) shows mixed apatite originating from in vivo growth and calcium carbonate that formed from laser damage during prior Raman analysis. Region (**B**) shows COM plates growing from COD dissolution. Region (**C**) shows an example of a large COM hexagonal plate. Region (**D**) Large COM crystal with apparent layering of organic matter. Maps for individual mineral components are shown in Figure S12.

Figure 5 shows a 1 µm spatial resolution map of a mixed oxalate and apatite stone (the process for building the overlayed map is described in the Supporting Information). Similar samples were studied in detail by Sivaguru et al.^6,7^ and we refer readers to their work for a more detailed discussion of stone formation mechanisms. This stone sample is notable for its relatively open structure with large, open pores among the crystals that were filled by cyanoacrylate during sample preparation. Several structures of interest are outlined in Fig. 5: mixed apatite and calcite (A), a COM crystal surrounded by a COD crystal (B), a large 100 µm scale hexagonal platelet of COM (C), and a cut single crystal of COM exhibiting layered growth (D). Calcium carbonate is rare in kidney stones and crystalluria^53,54^ indicating the calcite we observe is likely the result of laser damage during Raman imaging. Though calcium carbonate may also form during laser lithotripsy, evidence from low-resolution maps strongly suggest damage from the Raman laser (Figs. S11, S13). Calcium carbonate in the preliminary low-resolution map has a spectrum closer to that of amorphous calcium carbonate whereas spatially averaged spectra from Fig. 5 match more closely with calcite (Figure S13). Had calcium carbonate been present prior to Raman laser irradiation it likely would have converted to calcite over time or during initial grinding and polishing of the sample. This apparent phase change between maps suggests formation of amorphous calcium carbonate due to heating of calcium oxalate to > 400 °C^55,56^ followed by conversion to calcite. Thus presence of calcium carbonate as detected by Raman spectroscopy should be treated with caution.

The COM crystal in (B) is surrounded entirely by COD which has been observed previously^7^. The COD-COM transition typically requires aqueous solution^57,58^. This appears to be a diagenetic COD-COM transition at the surface of the COD crystal as the COM morphology. The appearance of layering is similar to in-vitro examples of COM growth on COD that we have observed in attempts at growing calcium oxalate stones (Figure S14). Region (C) shows a ≈ 100 µm long crystal with hexagonal plate morphology that can be induced by additives like citrate or by high ionic strength solutions which both inhibit growth perpedicular to the charged (100) planes of COM^59,60^.

The crystal in region (D) shows a cut perpendicular to the (010) axis^59^ and, despite having the morphology of a COM single crystal, shows layered variation with visually darker layers having greater autofluorescence (Figure S15). Such layering suggests crystal (D) crystals did not grow in a constant environment and may have grown through deposition of amorphous calcium oxalate^61,62^ that converted to COM upon contact with existing crystals leading to a single-crystal-like structure while allowing incorporation of protein.

### Mixed oxalates

**Figure 6:**
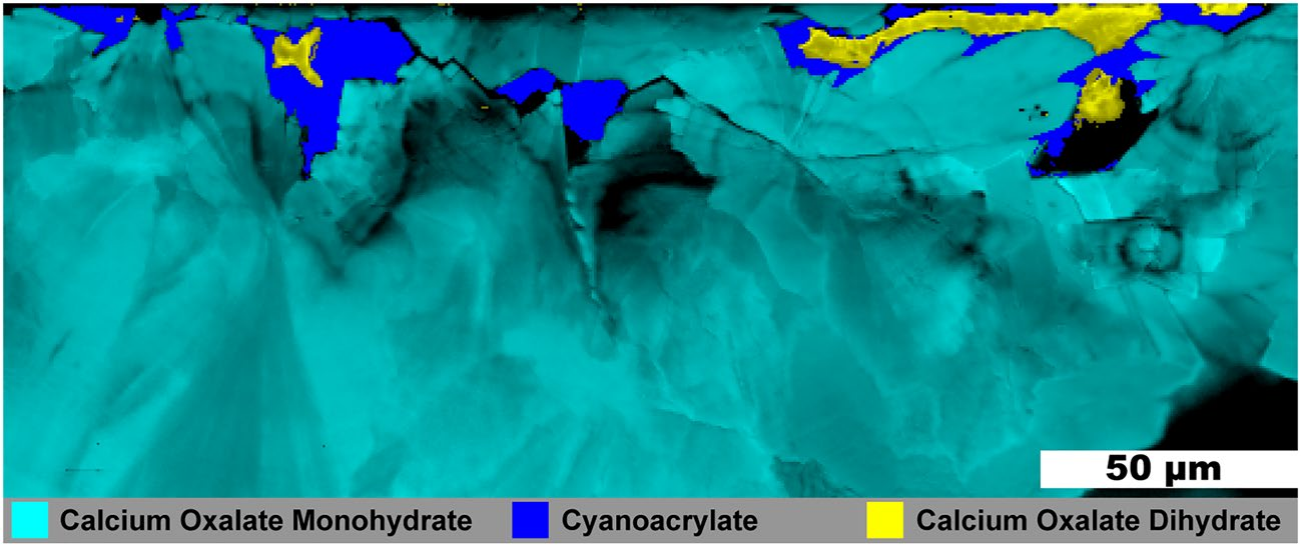
Raman image of a mixed calcium oxalate stone fragment. 0.5 µm spatial resolution map of calcium oxalate dihydrate and calcium oxalate monohydrate from a mixed calcium oxalate + apatite stone. White light image is shown in Figure S16.

Figure 6 shows another ~ 1 mm fragment from the same stone as that shown in Fig. 5. No HAp was observed in this fragment and much of the COM in Fig. 6 has a radial structure with layering. Three small, irregular veins of COD are present and all are surrounded by the cyanoacrylate glue used for embedding. As COM has a lower solubility than COD at room temperature, calcium and oxalate ions from dissolved COD appear to be reprecipitating on the radial COM bundles thereby replacing COM as described by Sivaguru^7^ The voids are left due to differences in density as COD (1.9 g/cm^3^) is less dense than the more stable COM (2.2 g/cm^3^).

### Struvite stone

**Figure 7:**
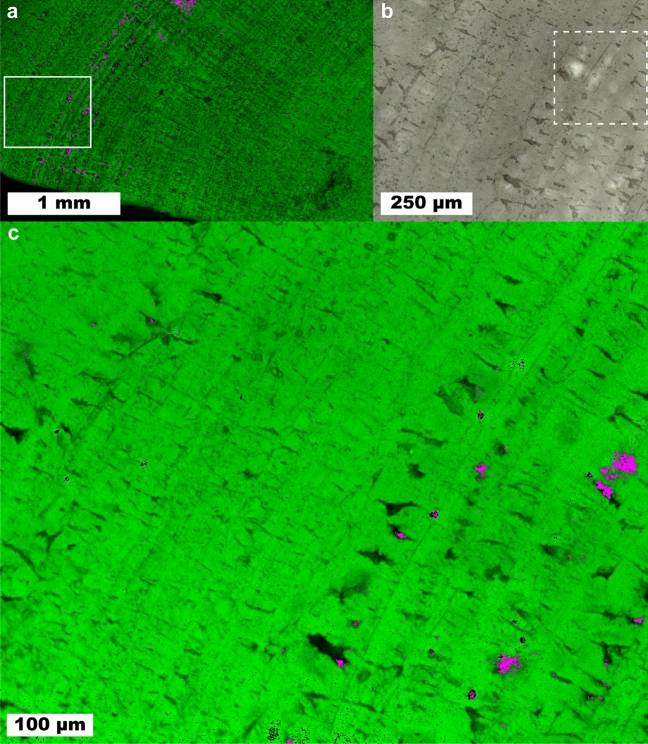
Raman mapping images of a predominantly struvite kidney stone. Small amounts of apatite (magenta) are interspersed among struvite (green). (**a**) 10 µm/pixel survey map of stone. (**b**) White light image of inset in (**a**). (**c**) Raman map of inset in (**a**).

A stone formed predominantly of struvite is imaged in Fig. 7. This stone shows a concentric layered structure suggesting even growth. The inset in Fig. 7a focuses on a region with mixed struvite and apatite. At the low irradiance settings used to avoid sample damage for struvite stones, these two minerals are difficult to distinguish as their most prominent peaks are centered at 945 and 962 cm^−1^ with full-width at half maximum of 21 and 11 cm^−1^ for each peak, respectively. Additionally, carbonate substitution in apatite structures shifts the phosphate ν_1_ peak to lower wavenumbers^63^ increasing the signal/noise required to resolve the two peaks. In lieu of higher irradiance, spatial averaging was used to confirm presence of apatite when signal/noise was too low in individual spectra.

Apatite was observed mainly near or within larger voids in the struvite layers. Unlike the case of calcium oxalates within “classic” brushite stones shown in Fig. 4, there is no clear difference in struvite morphology between regions containing apatite and those without. This suggests that the environment for struvite crystallization remained relatively constant during stone formation and that apatite either formed in urine and aggregated onto the growing struvite stone^64^ or formed by dissolution and conversion of struvite in the presence of calcium ions^65,66^. As with the COD-to-COM conversion, the voids could be formed during the dissolution and conversion process as hydroxyapatite (3.2 g/cm^3^) is more dense than struvite (1.7 g/cm^3^).

### Volume Raman mapping

**Figure 8:**
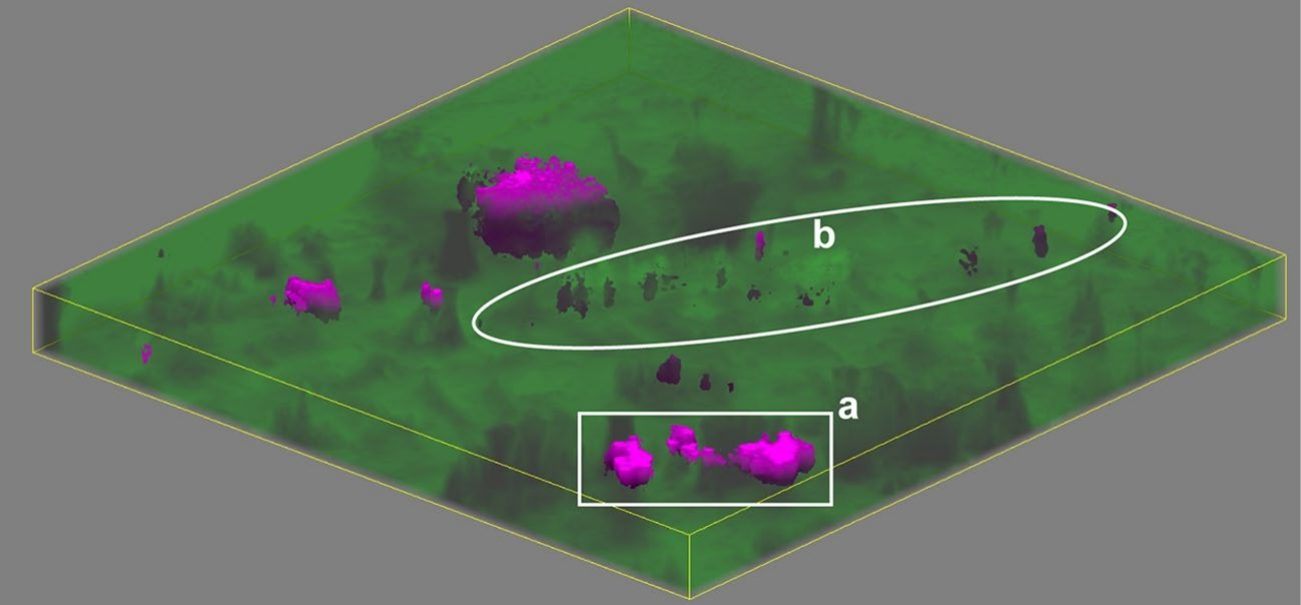
A volume map of a predominantly struvite stone. The area corresponds to the region marked in Fig. 7b. Raman volume imaging was conducted with a nominal resolution of 1 µm^3^ voxels. Struvite and apatite are represented by green and magenta, respectively, with higher saturation indicating a closer fit by least squares. Sides of the image are 250 µm long and depth is ≈ 13.3 µm for 20 µm of stage movement and the refractive index of struvite (≈ 1.5).

Figure 8 shows the results of confocal volume imaging the region marked by the white box in Fig. 7b. Volume imaging confirms the observation that apatite is associated with voids that are exposed at the surface of the struvite stone (Fig. 8, region a). Volume imaging also revealed the presence of sub-surface apatite in region (b) of Fig. 8 which appears as a relatively brighter band in optical images (Fig. 7b). Apatite in kidney stones typically takes the form of aggregated spherules^67^ with diameters on the order of 10 µm. We expect these small spherules are more effective at backscattering light in optical images than organized crystal growths because refractive index will vary on shorter length scales in the aggregated spherulites increasing opportunities reflection, multiple refraction, or other forms of scattering. Relating optical cues to chemical and structural features is potentially valuable for practical application by urologists as optical differences may help urologists focus on specific mineral types. Further testing with ex-vivo lithotripsy is required to understand specific relationships between optical cues and ablation behavior.

### Applying Raman mapping to lithotripsy research

Raman mapping and volume imaging have the potential to enable experiments that image kidney stone composition and structure before and after ex-vivo laser ablation of stones. While scattering by individual crystals within a kidney stone may prevent volume imaging to the ≈ 400 µm deep craters^68^ caused by 1 J laser pulses, surgical lasers operating in “dusting” mode or with a fiber standoff distance produce shallower features that can be fully imaged. One open question in laser lithotripsy is whether free urine or water within voids contributes significantly to stone ablation. Volume imaging Raman spectroscopy followed by laser ablation of sample stones has the potential to offer insight into this potential mechanism as mineral composition, void structure, and fluorescent molecule presence can be correlated to features of ablated particles and the craters that remain.

## Conclusion

We have demonstrated the ability of Raman microscopy to produce 0.5–1 µm spatial resolution chemical maps of kidney stone samples. Hydrogen peroxide combined with light was used to decrease autofluorescence enabling Raman spectroscopy without the need for extensive photobleaching. High-resolution chemical maps were used to identify potential mechanisms of kidney stone growth for the six most common stone minerals: COM, COD, HAp, uric acid, brushite, and struvite. Mapping a type 1a COM stone revealed COD agglomeration likely plays a role in disrupting concentric layer growth. In contrast, thin concentric layers of COM within uric acid stones were observed to form without disrupting the concentric layering of uric acid itself. This suggests nucleation of COM occurred at the surface of the uric acid stone, a potential example nucleation by pseudoseeding. A brushite stone with distinct regions of compact, layered growth or interlocking radial bundles showed greater presence of calcium oxalates among the void-prone, interlocking radial bundles of brushite. This suggested high calcium concentrations contributed to simultaneous precipitation of all three minerals. Observations of mixed COM, COD, and HAp stones show evidence for COD dissolution with subsequent recrystallization as COM. Large regions with COM single crystal morphologies exhibited apparent layering of organic material. Apatite in struvite stones was primarily found near or within void spaces suggesting formation during dissolution of struvite into more stable and denser apatitic forms. Volume mapping with 1 µm^3^ voxel resolution was performed to a depth of ≈13.3 µm on a struvite kidney stone for the first time.

With simultaneous structural and compositional analysis, Raman mapping can be used to understand both physical and chemical structure of kidney stones more completely than other techniques. Future directions for Raman imaging include observing chemical changes in both ablated sediment and exposed craters after laser ablation of previously mapped areas. Relationships between laser lithotripsy conditions and efficient stone destruction can then be identified and optimized to decrease time needed for surgery while avoiding thermal damage to surrounding tissues. With volume imaging, Raman microscopy can be used to study the importance of laser ablation mechanisms like the vaporization of urine within pores. The improvement in Raman spatial resolution allowed identification of previously unobserved features including ≈1 μm thick COM layers in uric acid stones and the co-localization of ≤ 5 μm calcium oxalate crystals with brushite crystals of specific, elongated morphology. Application of Raman imaging to a wider variety of stones will lead to new insights into kidney stone growth benefitting both scientists seeking to better understand pathological biomineralization and clinicians seeking to improve treatment of kidney stone disease.

## Methods

Kidney stone samples were selected from a de-identified library of human stones for Raman mapping. For polishing, smaller fragments (< 5 mm) were embedded in cyanoacrylate glue or CrystalBond 509 in depressions milled at the center of 1 inch outer diameter acrylic holders. Crystalbond 509 heated to 125 °C was preferred for minerals with higher decomposition temperatures (COM, UA, Hap) while cyanoacrylate was preferred for minerals that would decompose on contact with melted CrystalBond 509.^69,70^ Larger fragments were polished by embedding in CrystalBond 509 (COM, UA, HAp) or CrystalBond 555 (COD, brushite, struvite) to allow for dissolution and removal of the embedding medium after polishing. CrystalBond 555 was melted at a temperature of 70 °C before embedding, below the temperature at which significant decomposition is expected to occur for brushite, struvite, and COD.

Samples were leveled by hand using wetted sandpaper of progressively finer grits ending at grit size J2500 which has an average particle size of 5.5 µm. Sandpaper was placed on float glass to maintain a flat grinding surface. Water was used for wetting sandpaper for samples embedded in cyanoacrylate and CrystalBond 509. Light mineral oil was used for samples embedded in CrystalBond 555.

After grinding, samples were polished consecutively using aqueous alumina suspensions of 1 µm and 0.3 µm nominal diameter on a low-nap cloth (Alpha-A Cloth, Ted Pella) backed by float glass. For struvite and brushite samples embedded in CrystalBond 555, aqueous alumina suspensions were mixed with mineral oil which coated the water-soluble CrystalBond 555 thereby preventing its dissolution during polishing. Mineral oil from grinding and polishing was removed by soaking samples in hexanes or petroleum ether overnight followed by rinsing. Soaking and rinsing was repeated at least three times.

After the first polishing, and prior to Raman mapping, kidney stone samples containing predominantly inorganic minerals were treated with hydrogen peroxide and light to deactivate autofluorescent organic material using a method adapted from Yakubovskaya et al.^31^ Samples were covered with 6% H_2_O_2_ (aq), typically 1 mL, and placed in a petri dish to prevent evaporation. A high-power rectangular array of white LEDs (GS Vitec LT-V9-15) was placed 6 cm from the sample and irradiation was carried out for 1 h. After irradiation the sample was gently rinsed with deionized water and allowed to air dry. Samples were re-polished after peroxide treatment with 0.3 µm alumina suspension as before. We attempted treatment of uric acid samples in the same manner as the inorganic minerals but observed no reduction in autofluorescence. Uric acid fluoresces strongly at 532 nm though Raman signal can still be observed with sufficient exposure time. As uric acid is an antioxidant known to scavenge hydroxyl radicals in the body, chemical bleaching may not be effective on uric acid stones^71,72^.

Raman mapping was performed using Renishaw InVia microscopes. Samples containing predominantly inorganic minerals were mapped using a 532 nm laser. High confocality mode was used for better fluorescence rejection when using the 532 nm laser unless otherwise noted. Uric acid stones were mapped using a 785 nm laser as autofluorescence could not be reduced by the hydrogen peroxide + light treatment. Standard confocality mode was used for uric acid stones. Surface-following contour maps for each sample were generated by manually focusing on a minimum of four points in the flat-polished regions of the sample. For the highest spatial resolution maps of 0.5 µm for COM, COD, or HAp, typical scan parameters were 0.1 s exposure for a scan speed of 5 µm/s and a laser power of ≈50 mW at the sample with a spot diameter of ≈0.5 µm for a 50 × objective. The resulting power density is ≈20 W/µm^2^. For struvite and brushite samples laser power was lowered to ≈25 mW at the sample surface, exposure times were decreased to 0.05 µs, and scan speeds increased to 20 µm/s for a power density of 2.5 W/µm^2^. The lowered irradiance applied to brushite and struvite prevented laser-induced damage on all polished and peroxide-treated samples tested. Higher irradiance may be used successfully though performing initial mapping at lower irradiance to test for damage is recommended.

### Supplementary Information

Supplementary Information.

Supplementary Information.

## Data Availability

Raw images in the manuscript and supporting information are provided individually in the electronic supporting materials. Tabulated data for Raman spectra plotted in the manuscript and supporting information are provided as CSV files in the electronic supporting materials.

## References

[1] Timlin JA, Carden A, Morris MD (1999). Chemical microstructure of cortical bone probed by Raman Transects. Appl. Spectrosc..

[2] Carden A, Morris MD (2000). Application of vibrational spectroscopy to the study of mineralized tissues (review). J. Biomed. Opt.

[3] Gupta SD, Killenberger M, Tanner T, Rieppo L, Saarakkala S, Heikkilä J, Anttonen VJ, Finnilä MA (2021). Mineralization of dental tissues and caries lesions detailed with raman microspectroscopic imaging. Analyst.

[4] Wentrup-Byrne E, Armstrong CA, Armstrong RS, Collins BM (1997). Fourier transform Raman microscopic mapping of the molecular components in a human tooth. J. Raman Spectrosc..

[5] Castiglione V, Sacré P-Y, Cavalier E, Hubert P, Gadisseur R, Ziemons E (2018). Raman chemical imaging, a new tool in kidney stone structure analysis: Case-study and comparison to fourier transform infrared spectroscopy. PLoS ONE.

[6] Sivaguru M, Saw JJ, Wilson EM, Lieske JC, Krambeck AE, Williams JC, Romero MF, Fouke KW, Curtis MW, Kear-Scott JL, Chia N, Fouke BW (2021). Human kidney stones: A natural record of universal biomineralization. Nat. Rev. Urol..

[7] Sivaguru M, Saw JJ, Williams JC, Lieske JC, Krambeck AE, Romero MF, Chia N, Schwaderer AL, Alcalde RE, Bruce WJ, Wildman DE, Fried GA, Werth CJ, Reeder RJ, Yau PM, Sanford RA, Fouke BW (2018). Geobiology reveals how human kidney stones dissolve in vivo. Sci. Rep..

[8] Todorov LG, Sivaguru M, Krambeck AE, Lee MS, Lieske JC, Fouke BW (2022). GeoBioMed perspectives on kidney stone recurrence from the reactive surface area of SWL-derived particles. Sci. Rep..

[9] Zarse CA, McAteer JA, Sommer AJ, Kim SC, Hatt EK, Lingeman JE, Evan AP, Williams JC (2004). Nondestructive analysis of urinary calculi using micro computed tomography. BMC Urol..

[10] Manzoor MAP, Agrawal AK, Singh B, Mujeeburahiman M, Rekha P-D (2019). Morphological characteristics and microstructure of kidney stones using synchrotron radiation μCT reveal the mechanism of crystal growth and aggregation in mixed stones. PLOS ONE.

[11] Corrales M, Doizi S, Barghouthy Y, Traxer O, Daudon M (2021). Classification of stones according to Michel Daudon: A narrative review. Eur. Urol. Focus.

[12] Williams JC, Lingeman JE, Daudon M, Bazin D (2021). Using micro computed tomographic imaging for analyzing kidney stones. Comptes Rendus. Chimie.

[13] Williams JC, Lingeman JE, Coe FL, Worcester EM, Evan AP (2015). Micro-CT imaging of Randall’s plaques. Urolithiasis.

[14] Williams JC, McAteer JA, Evan AP, Lingeman JE (2010). Micro-computed tomography for analysis of urinary calculi. Urol. Res..

[15] Williams JC, Worcester E, Lingeman JE (2017). What can the microstructure of stones tell us?. Urolithiasis.

[16] Keller EX, de Coninck V, Audouin M, Doizi S, Bazin D, Daudon M, Traxer O (2019). Fragments and dust after holmium laser lithotripsy with or without “Moses technology”: How are they different?. J. Biophotonics.

[17] Daudon M, Bazin D, André G, Jungers P, Cousson A, Chevallier P, Véron E, Matzen G (2009). Examination of Whewellite kidney stones by scanning electron microscopy and powder neutron diffraction techniques. J. Appl. Crystallogr..

[18] Al-Atar U, Bokov AA, Marshall D, Teichman JMH, Gates BD, Ye Z-G, Branda NR (2010). Mechanism of calcium oxalate monohydrate kidney stones formation: Layered spherulitic growth. Chem. Mater..

[19] Gleeson M, Morizet J, Mahou P, Daudon M, Bazin D, Stringari C, Schanne-Klein M-C, Beaurepaire E (2023). Kidney stone classification using multimodal multiphoton microscopy. ACS Photonics.

[20] Anderson JC, Williams JC, Evan AP, Condon KW, Sommer AJ (2007). Analysis of urinary calculi using an infrared microspectroscopic surface reflectance imaging technique. Urol. Res..

[21] Bazin D, Jouanneau C, Bertazzo S, Sandt C, Dessombz A, Réfrégiers M, Dumas P, Frederick J, Haymann J-P, Letavernier E, Ronco P, Daudon M (2016). Combining field effect scanning electron microscopy, deep UV fluorescence, Raman, classical and synchrotron radiation fourier transform infra-red spectroscopy in the study of crystal-containing kidney biopsies. Comptes Rendus Chimie.

[22] Valido H, Resina-Gallego I, Yousef M, Luque-Gálvez I, Valiente MP, López-Mesas M (2020). Calcium oxalate kidney stones, where is the organic matter?: A synchrotron based infrared microspectroscopy study. J. Biophotonics.

[23] Sofińska-Chmiel W, Goliszek M, Drewniak M, Nowicka A, Kuśmierz M, Adamczuk A, Malinowska P, Maciejewski R, Tatarczak-Michalewska M, Blicharska E (2023). Chemical studies of multicomponent kidney stones using the modern advanced research methods. Molecules.

[24] Daudon M, Protat MF, Reveillaud RJ, Jaeschke-Boyer H (1983). Infrared spectrometry and Raman microprobe in the analysis of urinary calculi. Kidney Int..

[25] Cloutier J, Villa L, Traxer O, Daudon M (2015). Kidney stone analysis: “Give Me Your Stone, I Will Tell You Who You Are!”. World J. Urol..

[26] Estepa L, Daudon M (1997). Contribution of Fourier transform infrared spectroscopy to the identification of urinary stones and kidney crystal deposits. Biospectroscopy.

[27] Tamosaityte S, Pucetaite M, Zelvys A, Varvuolyte S, Hendrixson V, Sablinskas V (2022). Raman spectroscopy as a non-destructive tool to determine the chemical composition of urinary sediments. Comptes Rendus. Chimie.

[28] Lucas IT, Bazin D, Daudon M (2022). Raman opportunities in the field of pathological calcifications. Comptes Rendus. Chimie.

[29] Cui X, Zhao Z, Zhang G, Chen S, Zhao Y, Lu J (2018). Analysis and classification of kidney stones based on Raman spectroscopy. Biomed. Opt. Express..

[30] Golcuk K, Mandair GS, Callender AF, Sahar N, Kohn DH, Morris MD (2006). Is photobleaching necessary for Raman imaging of bone tissue using a green laser?. Biochim. Biophys. Acta Biomembr..

[31] Yakubovskaya E, Zaliznyak T, Martínez Martínez J, Taylor GT (2019). Tear down the fluorescent curtain: A new fluorescence suppression method for Raman microspectroscopic analyses. Sci. Rep..

[32] Petit I, Belletti GD, Debroise T, Llansola-Portoles MJ, Lucas IT, Leroy C, Bonhomme C, Bonhomme-Coury L, Bazin D, Daudon M, Letavernier E, Haymann JP, Frochot V, Babonneau F, Quaino P, Tielens F (2018). Vibrational signatures of calcium oxalate polyhydrates. ChemistrySelect.

[33] Ulian G, Valdrè G, Corno M, Ugliengo P (2013). The vibrational features of hydroxylapatite and Type A carbonated apatite: A first principle contribution. Am. Mineralogist.

[34] Casciani F, Condrate RA (1979). The vibrational spectra of brushite, CaHPO_4_·2H_2_O. Spectrosc. Lett..

[35] Stefov V, Šoptrajanov B, Kuzmanovski I, Lutz HD, Engelen B (2005). Infrared and Raman spectra of magnesium ammonium phosphate hexahydrate (struvite) and its isomorphous analogues. III. Spectra of protiated and partially deuterated magnesium ammonium phosphate hexahydrate. J. Mol. Struct..

[36] Zellelow AZ, Kim K-H, Sours RE, Swift JA (2010). Solid-state dehydration of uric acid dihydrate. Cryst. Growth Des..

[37] Izatulina AR, Gurzhiy VV, Krzhizhanovskaya MG, Chukanov NV, Panikorovskii TL (2019). Thermal behavior and phase transition of uric acid and its dihydrate form, the common biominerals uricite and tinnunculite. Minerals.

[38] Presores JB, Swift JA (2014). Solution-mediated phase transformation of uric acid dihydrate. CrystEngComm.

[39] Frincu MC, Fogarty CE, Swift JA (2004). Epitaxial relationships between uric acid crystals and mineral surfaces: A factor in urinary stone formation. Langmuir.

[40] Wang Z, Königsberger E (1998). Solubility equilibria in the uric acid-sodium urate–water system. Thermochim. Acta.

[41] Königsberger E, Tromans A, May PM, Hefter G (2021). Solubility of calcium oxalate monohydrate in concentrated electrolyte solutions. J. Chem. Eng. Data.

[42] Ibis F, Dhand P, Suleymanli S, van der Heijden AEDM, Kramer HJM, Eral HB (2020). A combined experimental and modelling study on solubility of calcium oxalate monohydrate at physiologically relevant pH and temperatures. Crystals.

[43] Gridley CM, Sourial MW, Lehman A, Knudsen BE (2019). Medical dissolution therapy for the treatment of uric acid nephrolithiasis. World J. Urol..

[44] Vermeulen CW, Fried FA (1965). Observations on dissolution of uric acid calculi. J. Urol..

[45] Pramanik R, Asplin JR, Jackson ME, Williams JC (2008). Protein content of human apatite and brushite kidney stones: Significant correlation with morphologic measures. Urol. Res..

[46] Brown WE, Patel PR, Chow LC (1975). Formation of CaHPO_4_ 2H_2_O from enamel mineral and its relationship to caries mechanism. J. Dent. Res..

[47] Brown PW (1992). Phase relationships in the ternary system CaO─P_2_O_5_─H_2_O at 25°C. J. Am. Ceram. Soc..

[48] Abbona F, Christensson F, Angela MF, Madsen HEL (1993). Crystal habit and growth conditions of brushite, CaHPO_4_⋅2H_2_O. J. Cryst. Growth.

[49] Siener R, Netzer L, Hesse A (2013). Determinants of brushite stone formation: A case-control study. PLOS ONE.

[50] Bazin D, Leroy C, Tielens F, Bonhomme C, Bonhomme-Coury L, Damay F, Le Denmat D, Sadoine J, Rode J, Frochot V, Letavernier E, Haymann J-P, Daudon M (2016). Hyperoxaluria Is related to whewellite and hypercalciuria to weddellite: What happens when crystalline conversion occurs?. Comptes Rendus Chimie.

[51] Sarigul N, Korkmaz F, Kurultak İ (2019). A new artificial urine protocol to better imitate human urine. Sci. Rep..

[52] Daudon M, Jungers P, Bazin D, Williams JC (2018). Recurrence rates of urinary calculi according to stone composition and morphology. Urolithiasis.

[53] Werness PG, Bergert JH, Smith LH (1981). Crystalluria. J. Cryst. Growth.

[54] Frochot V, Castiglione V, Lucas IT, Haymann J-P, Letavernier E, Bazin D, Fogazzi GB, Daudon M (2021). Advances in the identification of calcium carbonate urinary crystals. Clin. Chim. Acta.

[55] Hourlier D (2019). Thermal decomposition of calcium oxalate: Beyond appearances. J. Therm. Anal. Calorim..

[56] Rak J, Skurski P, Gutowski M, Błażejowski J (1995). Thermodynamics of the thermal decomposition of calcium oxalate monohydrate examined theoretically. J. Therm. Anal. Calorim..

[57] Izatulina AR, Gurzhiy VV, Krzhizhanovskaya MG, Kuzmina MA, Leoni M, Frank-Kamenetskaya OV (2018). Hydrated calcium oxalates: Crystal structures, thermal stability, and phase evolution. Cryst. Growth Des..

[58] Echigo T, Kimata M, Kyono A, Shimizu M, Hatta T (2005). Re-investigation of the crystal structure of whewellite [Ca(C_2_O_4_)·H_2_O] and the dehydration mechanism of caoxite [Ca(C_2_O_4_)·3H_2_O]. Mineralogical Mag..

[59] Millan A (2001). Crystal growth shape of whewellite polymorphs: Influence of structure distortions on crystal shape. Cryst. Growth Des..

[60] Robinson JW, Ghani KR, Roberts WW, Matzger AJ (2023). Near-infrared absorption coefficients in kidney stone minerals and their relation to crystal structure. J. Phys. Chem. C.

[61] Hajir M, Graf R, Tremel W (2014). Stable amorphous calcium oxalate: Synthesis and potential intermediate in biomineralization. Chem. Commun..

[62] Ihli J, Wang Y-W, Cantaert B, Kim Y-Y, Green DC, Bomans PHH, Sommerdijk NAJM, Meldrum FC (2015). Precipitation of amorphous calcium oxalate in aqueous solution. Chem. Mater..

[63] Awonusi A, Morris MD, Tecklenburg MMJ (2007). Carbonate assignment and calibration in the Raman spectrum of apatite. Calcif. Tissue Int..

[64] Prywer J, Sadowski RR, Torzewska A (2015). Aggregation of struvite, carbonate apatite, and proteus mirabilis as a key factor of infectious urinary stone formation. Cryst. Growth Des..

[65] Qin L, Putnis CV, Wang L (2021). Facet-specific dissolution-precipitation at struvite-water interfaces. Cryst. Growth Des..

[66] Kurtulus G, Tas AC (2011). Transformations of neat and heated struvite (MgNH_4_PO_4_⋅6H_2_O). Mater. Lett..

[67] Racek M, Racek J, Hupáková I (2019). Scanning electron microscopy in analysis of urinary stones. Scand. J. Clin. Lab. Investig..

[68] Frank DS, Aldoukhi AH, Roberts WW, Ghani KR, Matzger AJ (2019). Polymer-mineral composites mimic human kidney stones in laser lithotripsy experiments. ACS Biomater. Sci. Eng..

[69] Frost RL, Weier ML, Erickson KL (2004). Thermal decomposition of struvite. J. Therm. Anal. Calorim..

[70] Bayuseno AP, Schmahl WW (2020). thermal decomposition of struvite in water: Qualitative and quantitative mineralogy analysis. Environ. Technol..

[71] Ames BN, Cathcart R, Schwiers E, Hochstein P (1981). uric acid provides an antioxidant defense in humans against oxidant- and radical-caused aging and cancer: A hypothesis. Proc. Natl. Acad. Sci..

[72] Simic MG, Jovanovic SV (1989). Antioxidation mechanisms of uric acid. J. Am. Chem. Soc..

