# Supplementary material for: Kidney stone growth through the lens of Raman mapping
Source: Sci Rep. 2024 May 12;14:10834. doi: 10.1038/s41598-024-61652-9 (PMC11088632; doi:10.1038/s41598-024-61652-9)
Supplement: Supplementary file 1 — Supplementary Information. [file 41598_2024_61652_MOESM1_ESM.pdf]

Kidney Stone Growth through the lens of Raman Mapping

Supporting Information

John W. Robinson, William W. Roberts, and Adam J. Matzger

Contents

Overlayed map construction	S2
Table S2: Raman settings for each map	S3
Figures S1-S7: Raman spectra for each powdered mineral	S4-S8
Figure S8: COM spectra with either the 1464 cm^{-1} and 1490 cm^{-1} peak dominant	S9
Figure S9: COM stone fit with Raman spectra of powdered minerals vs. oriented COM	S10
Figure S10: White light images for brushite stone in Figure 4.	S11-S12
Figure S11: White light image for Figure 5	S13
Figure S12: Individual component maps of Figure 5	S13
Figure S13: Comparison of calcium carbonate spectra	S14
Figure S14: SEM image of hexagonal COM plates growing on COD	S15
Figure S15: Spectra comparison for dark and light regions of crystal in Figure 5, region C	S16
Figure S16: White light image for map in Figure 6	S17

Mixed-phase false-color maps

False-color maps of mixed COD, COM, HAp, calcite, and cyanoacrylate were constructed using the Mapping Review function of Renishaw's WiRE software (v5.6). The non-negative least squares fit produces a Map value for each component with higher numbers indicating better fit. For each component, pixels below its ≈20th percentile map value were set to be transparent. The multi-component maps were constructed by layering each component on a black background. For Figure 5, the layer order is calcite (bottom), hydroxyapatite, cyanoacrylate, COM, and COD (top). This means, or areas of mixed COD/COM, that COD will cover COM and often appears as a darker yellow as map value of COD decreases as the map value for COM increases.

For Figure 6, the layer order is COM (bottom), cyanoacrylate, and COD (top).

In Figures 4 and 7 minor components (oxalates, apatites) were layered on top of dominant minerals (brushite, struvite).

Figure	Major minerals	Laser λ	Spatial resolution	Objective	Exposure time (s)	Confocality
2a, 2c	COM	532 nm	5 µm	50x	0.1	High
2b,d,f	COM	532 nm	1 µm	50x	0.5	High
3a,b	Uric acid	785 nm	10 µm	20x	1	Standard
3d,e	Uric acid	785 nm	1 µm	50x	1	Standard
4a	Brushite	532 nm	10 µm	50x	0.05	High
4b	Brushite	532 nm	1 µm	50x	0.025	High
4c	Brushite	532 nm	1 µm	50x	0.05	Standard
5	COD, COM, HAp	532 nm	0.5 µm	50x	0.1	High
6	COD, COM	532 nm	0.5 µm	100x	0.5	High
7a	Struvite	532 nm	10 µm	50x	0.05	Standard
7b	Struvite	532 nm	1 µm	50x	0.05	Standard
8	Struvite	532 nm	1 µm (voxel)	50x	0.05	High

Table S1: Raman collection settings for each map presented.

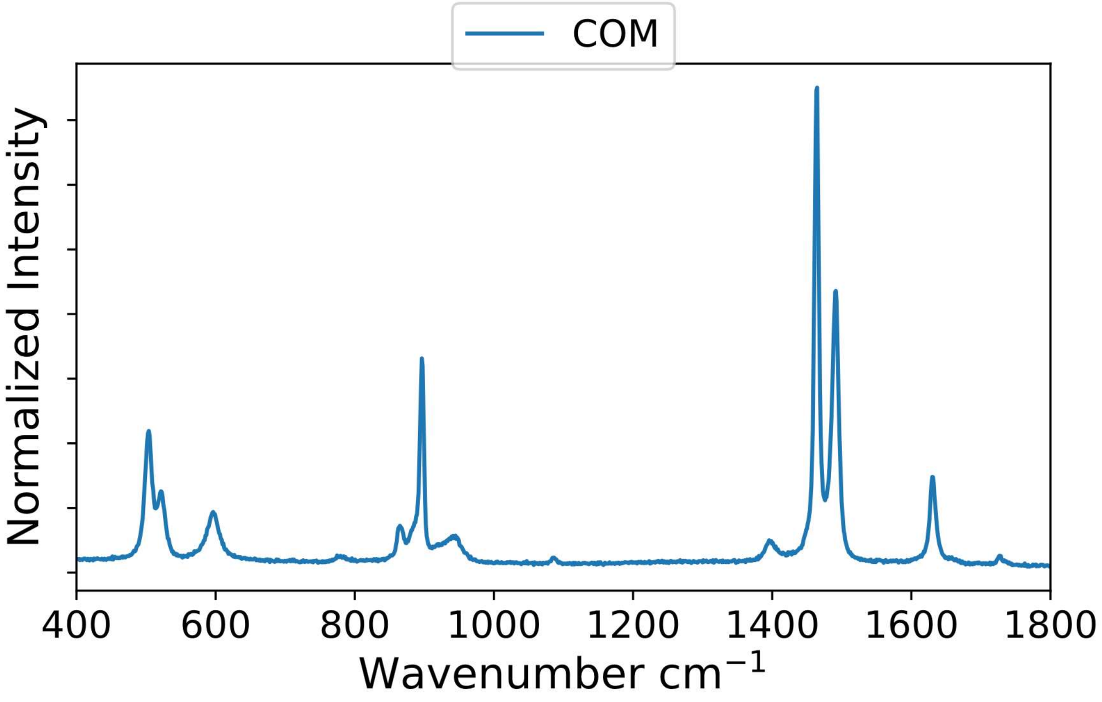

Figure S1: Raman spectrum of calcium oxalate monohydrate powder from 400-1800 cm^{-1} using 532 nm laser.

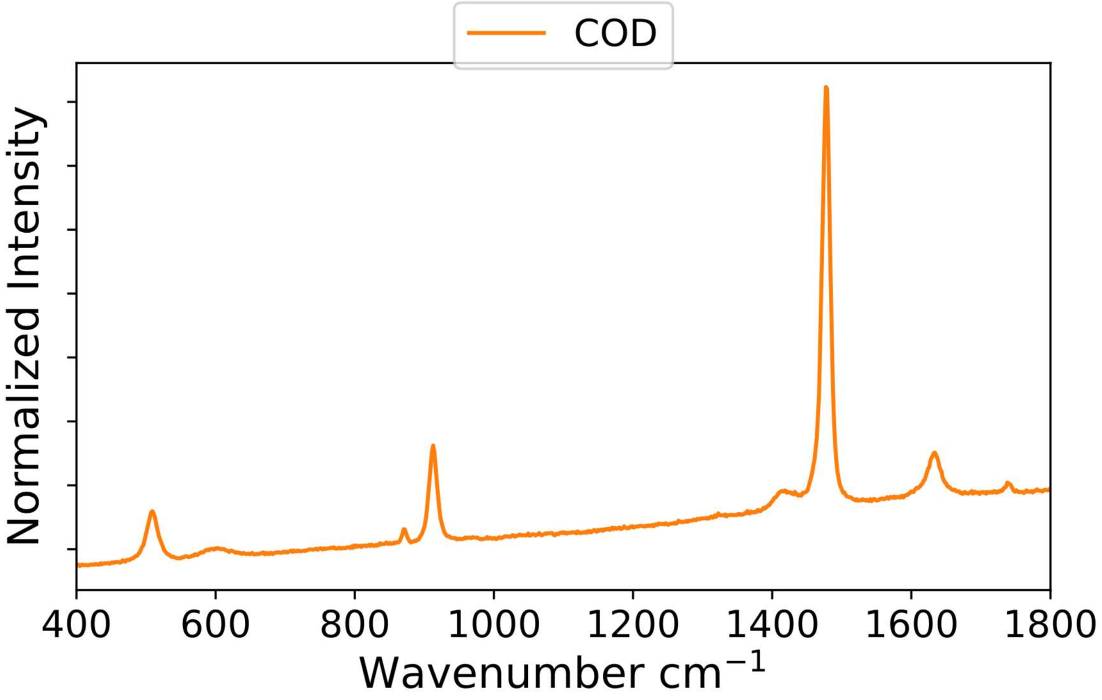

Figure S2: Raman spectrum from calcium oxalate dihydrate powder from 400-1800 cm^{-1} using 532 nm laser.

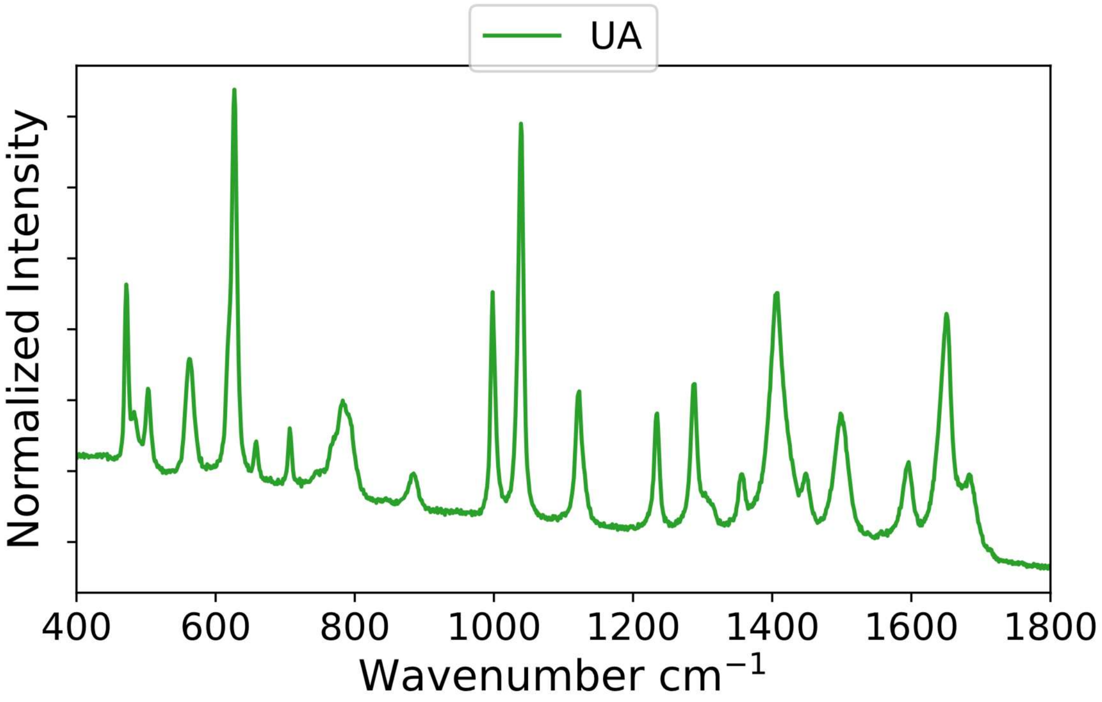

Figure S3: Raman spectrum of uric acid powder from 400-1800 cm^{-1} using 785 nm laser.

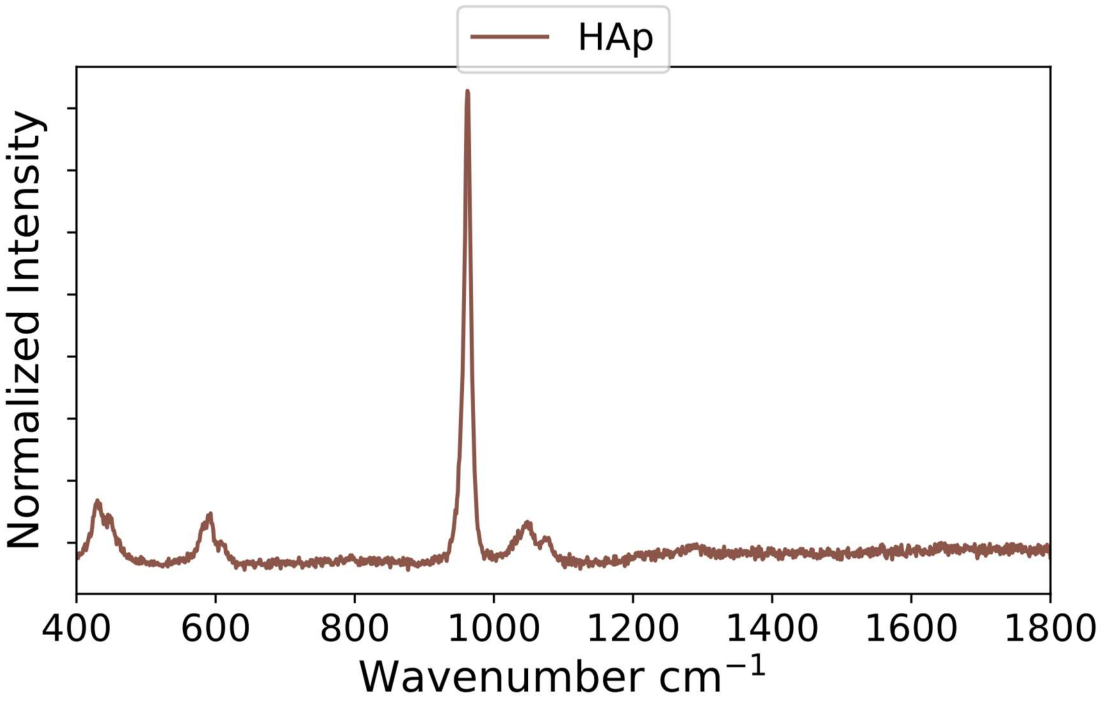

Figure S4: Raman spectrum of hydroxyapatite powder from 400-1800 cm^{-1} using 532 nm laser.

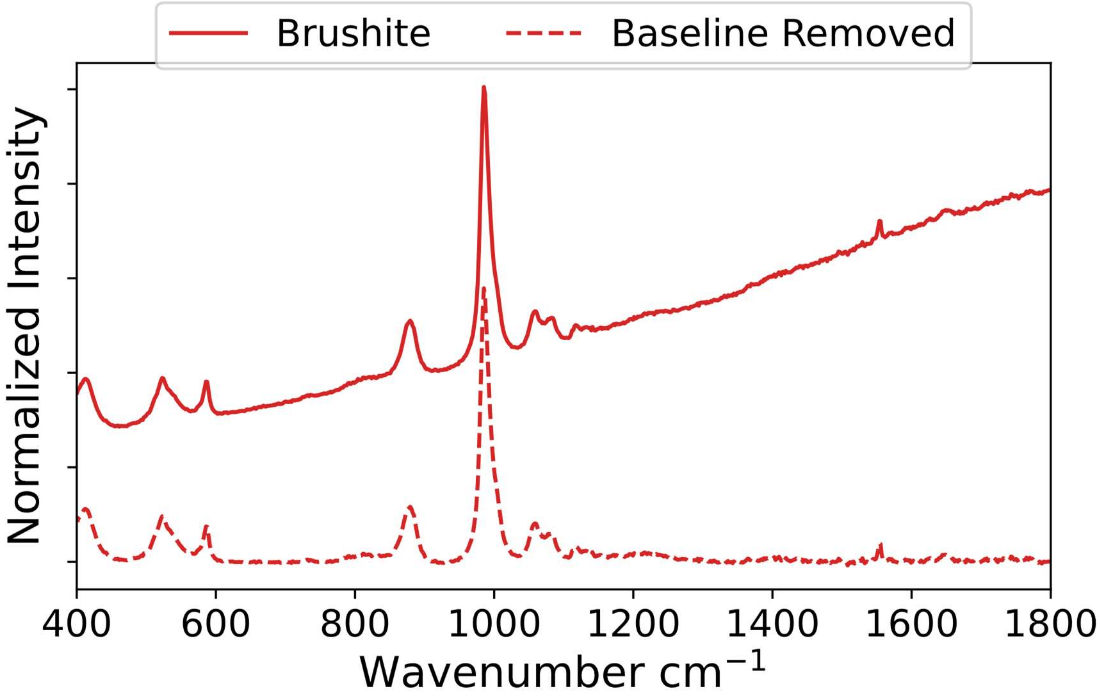

Figure S5: Raman spectrum of brushite powder from 400-1800 cm^{-1} using 532 nm laser. Dashed curve shows spectrum with fluorescence baseline removed.

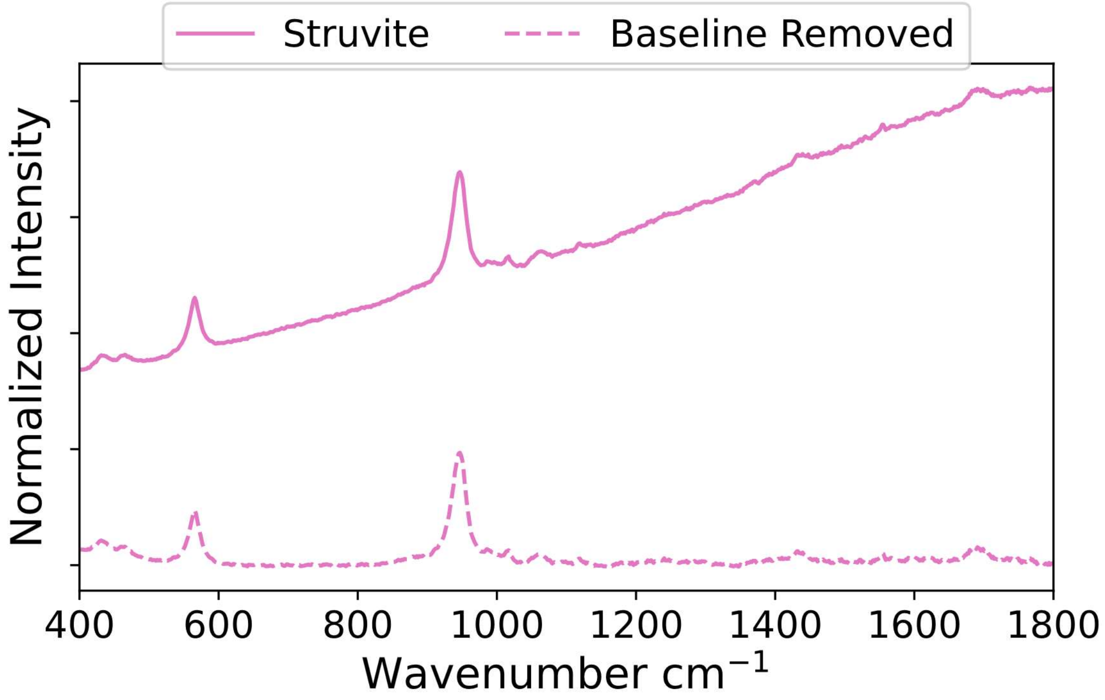

Figure S6: Raman spectrum of struvite powder from 400-1800 cm^{-1} using 532 nm laser. Dashed curve shows spectrum with fluorescence baseline removed.

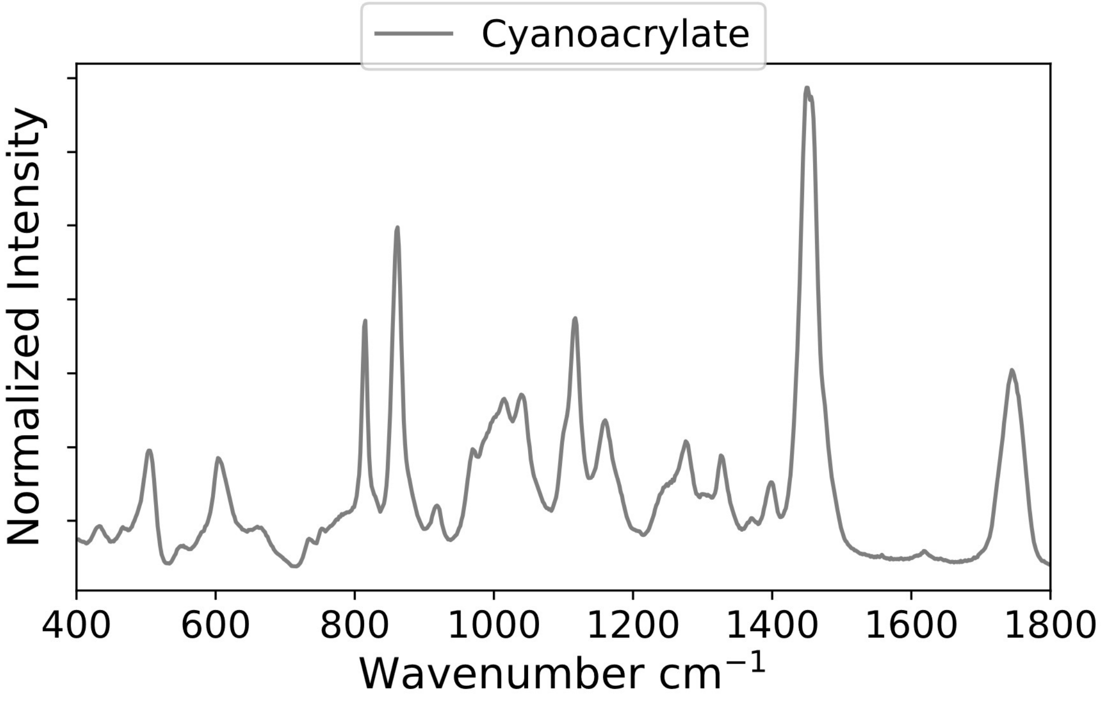

Figure S7: Raman spectrum of cyanoacrylate from 400-1800 cm^{-1} using 532 nm laser. Cyanoacrylate was clear and had cured at room temperature for 1 week.

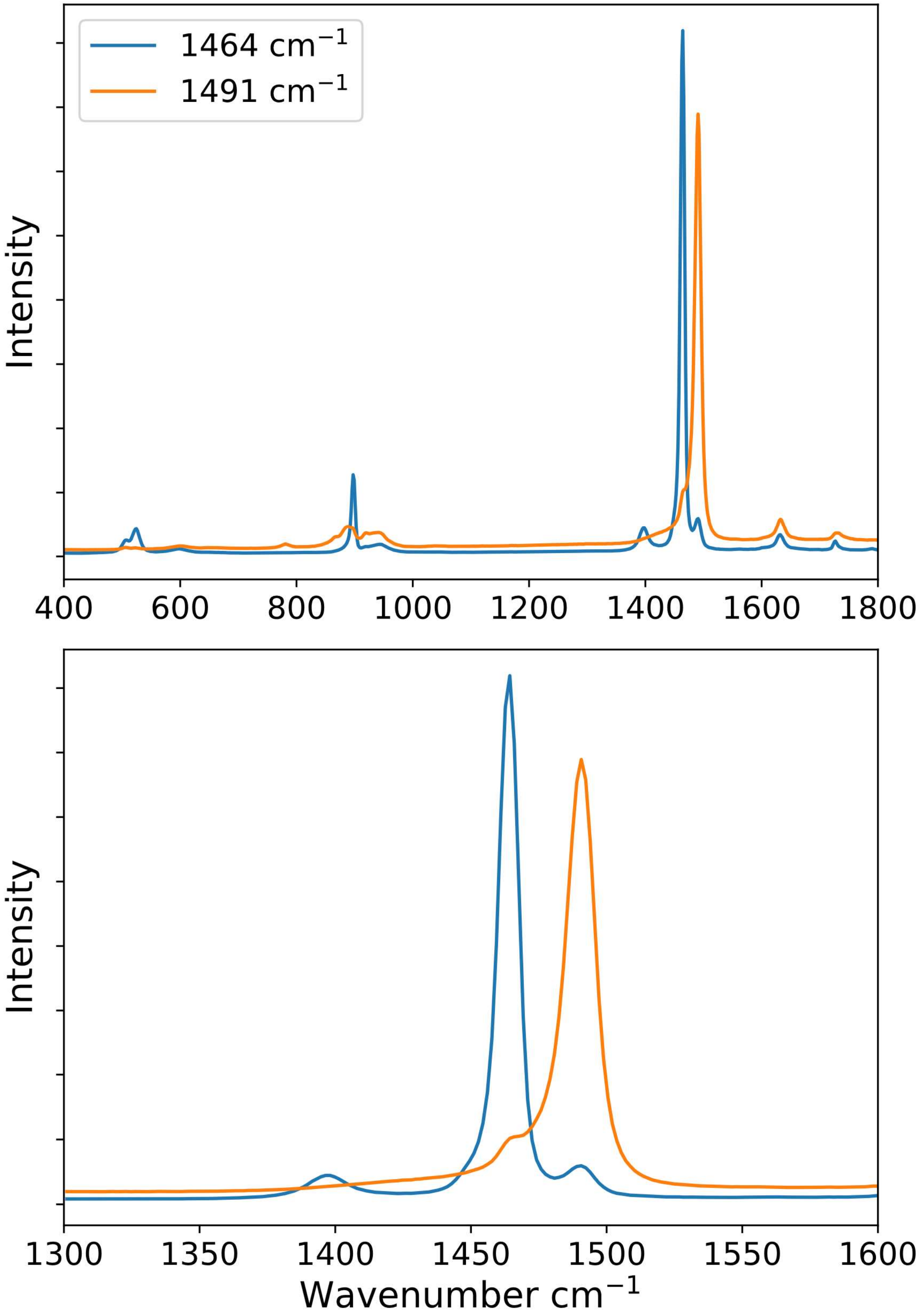

Figure S8: Raman spectrum of COM crystal in different orientations.

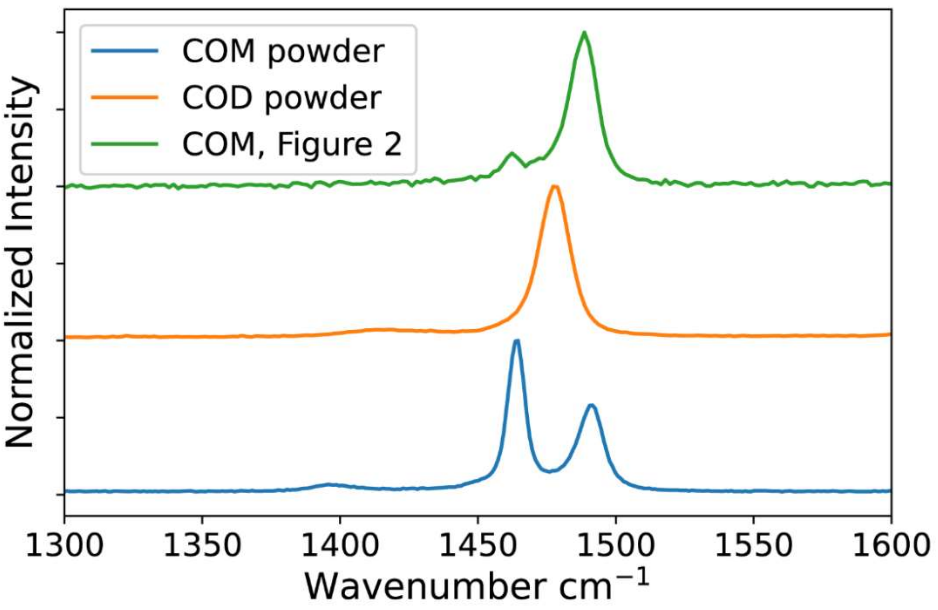

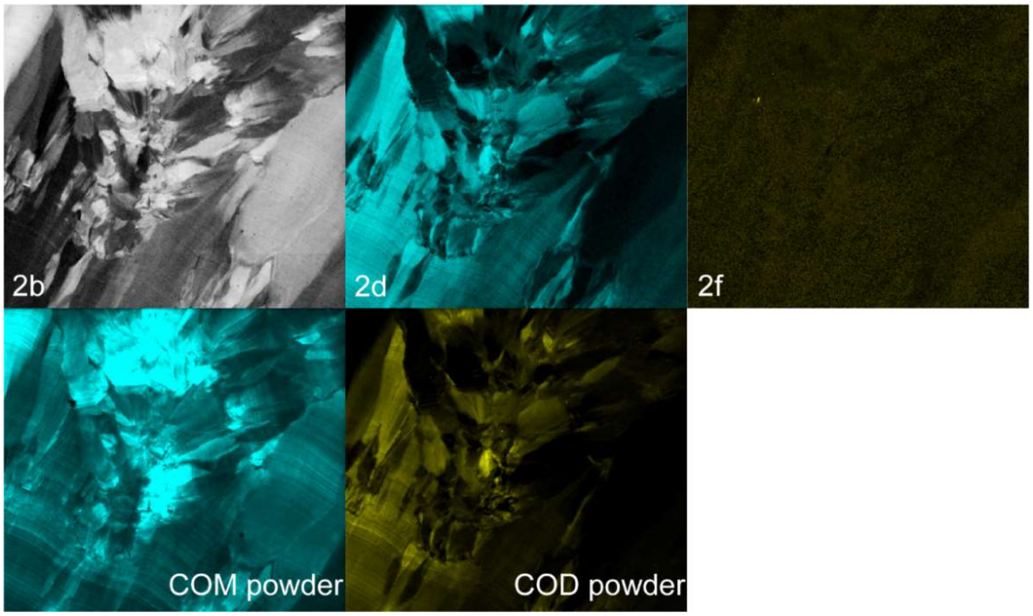

Figure S9: Top: overlayed spectra of COM powder, COD powder, and oriented COM spectrum from Figure 2.Bottom images: 2b, 2d, and 2f as in Figure 2. COM powder and COD powder: least-squares fit map using Raman spectra from powdered minerals.

For the oriented COM spectrum (green) in the top plot, the fraction of COD present was estimated to be 13% when least-squares fitting with separate spectra for COM as in Figure S8. When using Raman spectra from powdered COD and COM, COD fraction was 42%. All spectra used for the least-squares fit were normalized to a mean variance of 1. COD appears to be identified due overlap between the COM peaks at 1464 and 1490 cm^{-1}. However, in most spectra identified as COD its minor peaks at 913 and 509 cm^{-1} are not present while minor peaks for COM at 897 and 864 cm^{-1} are visible when the carboxylate stretch at 1490 cm^{-1} dominates making it likely that only COM is present.

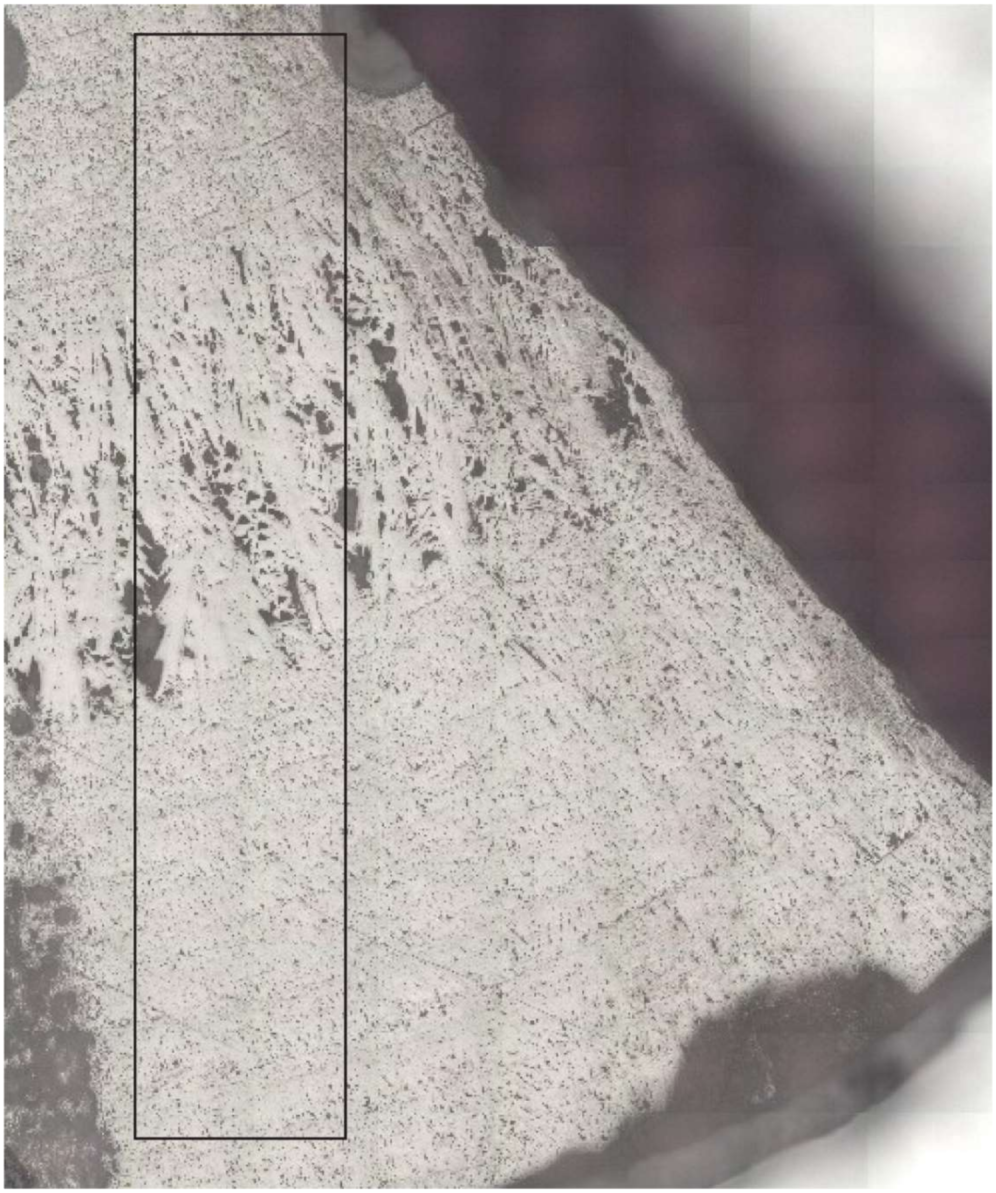

Figure S10a: White light image for brushite stone in Figure 4a. Box indicates area mapped.

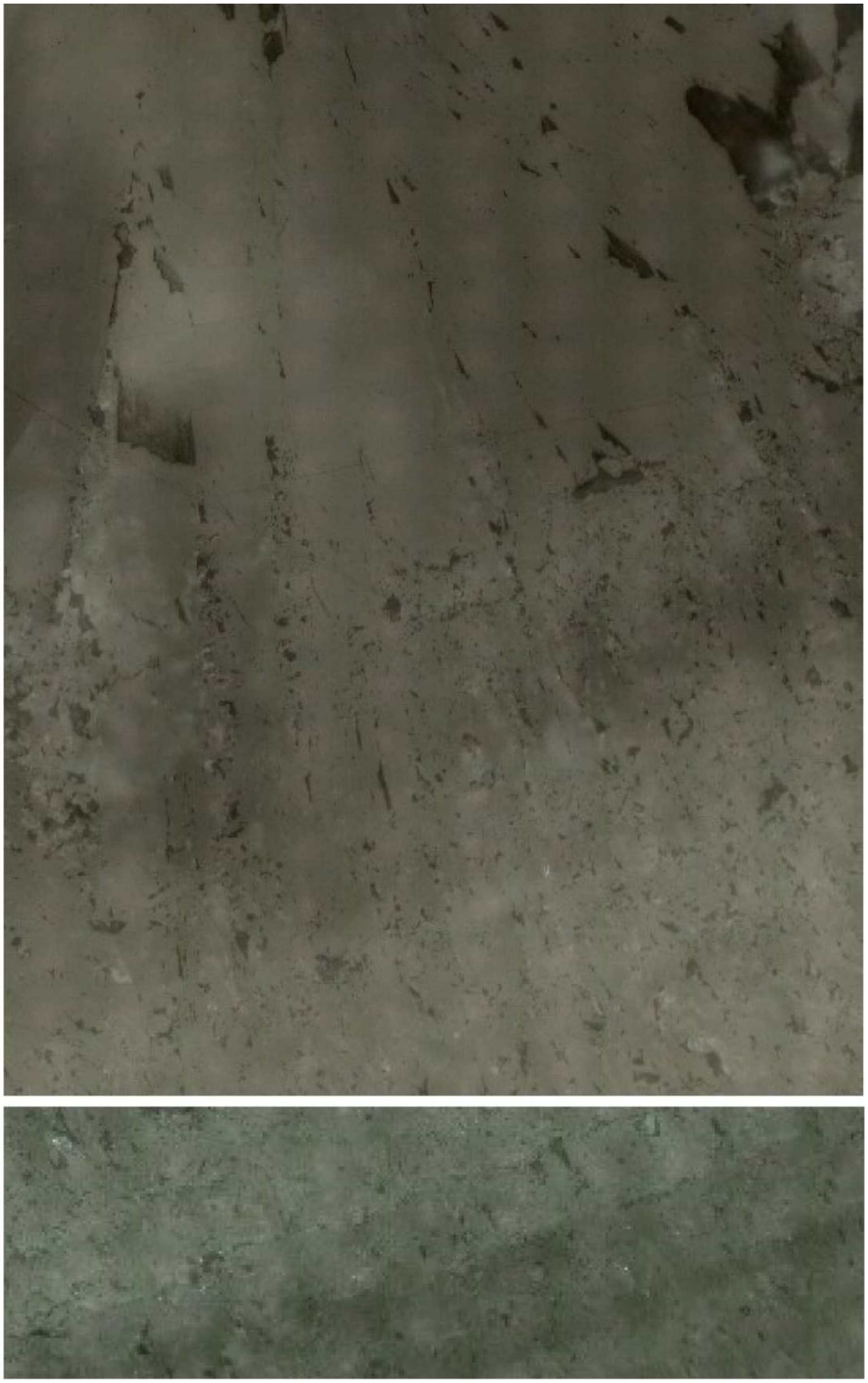

Figure S10b: White light images for Figure 4b and 4c.

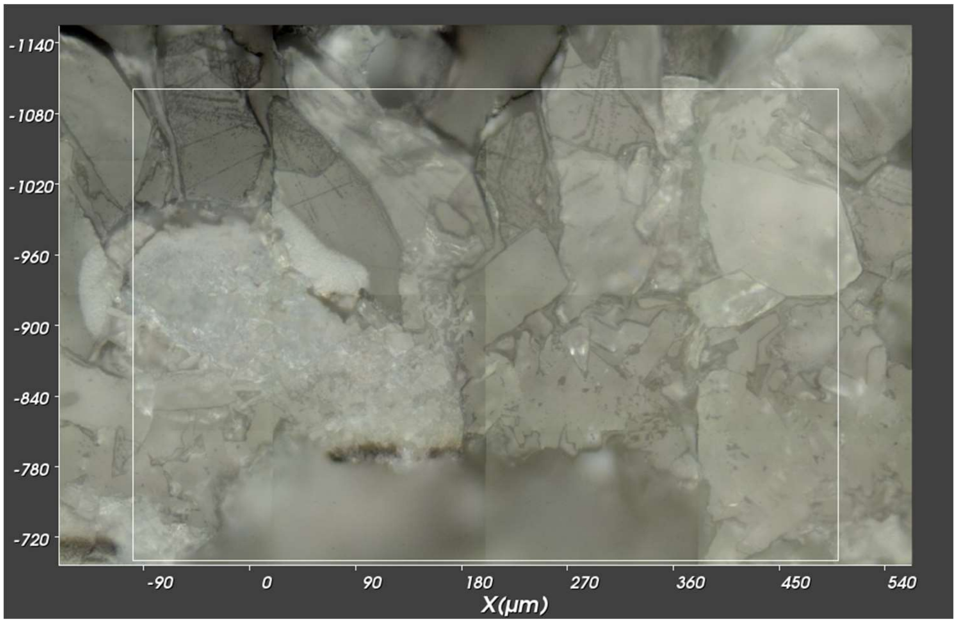

Figure S11: White light image of area mapped in Figure 5. Earlier laser damage is indicated by blackened areas where protein was carbonized.

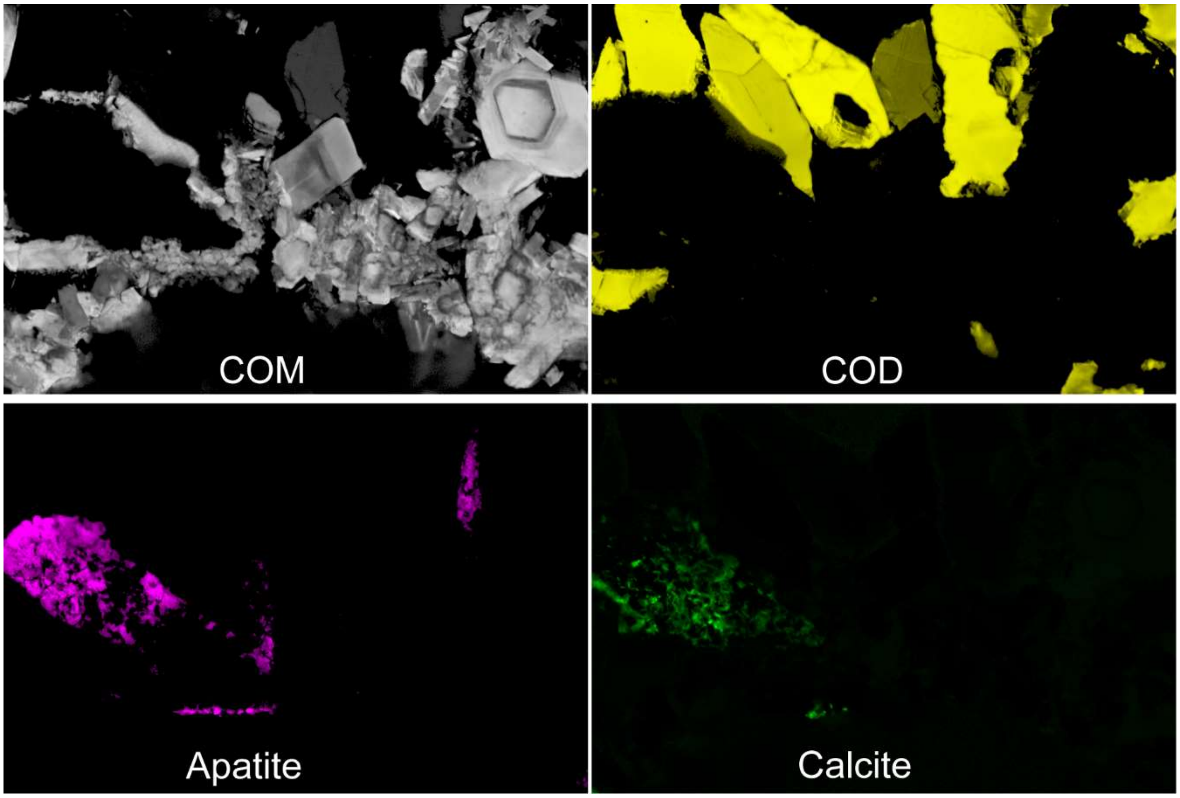

Figure S12: Individual component maps used to construct Figure 5 (excluding cyanoacrylate). Pure black (RGB values of 0,0,0) areas were made transparent for all but the bottom layer (calcite) to achieve overlay effect.

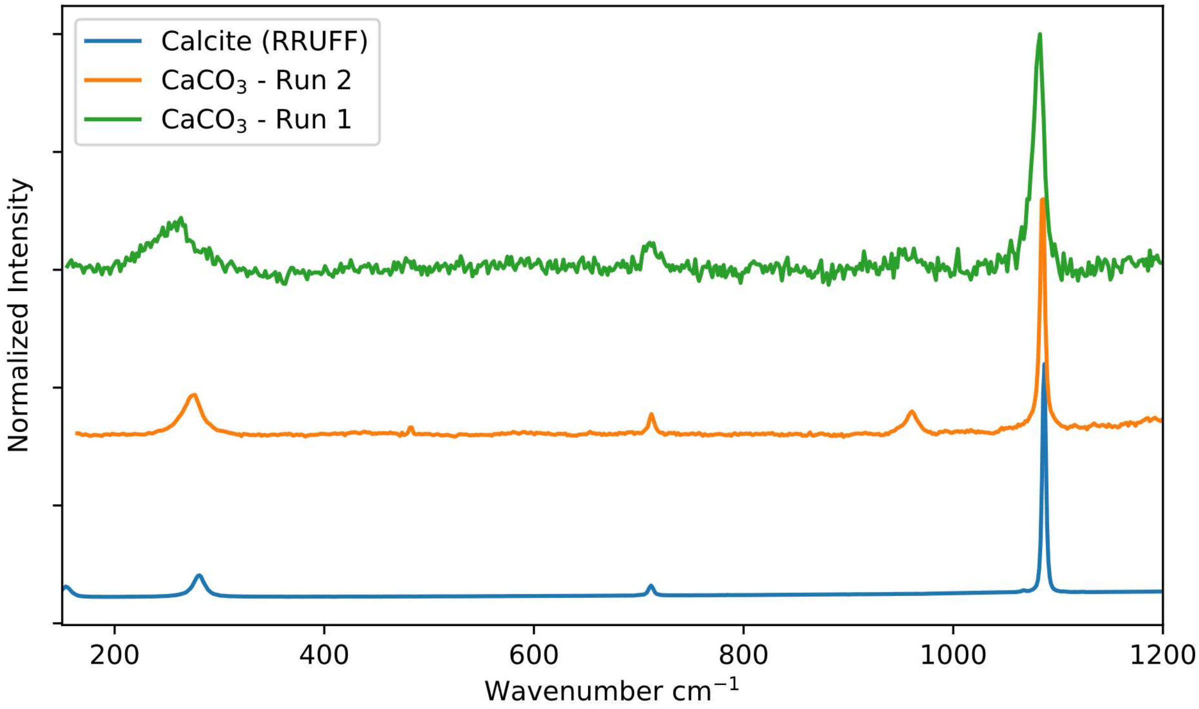

Figure S13: Comparison of calcium carbonate from an initial survey run (green) and high-resolution map (orange) compared to calcite from the RRUFF database (blue) for sample shown in Figure 5 and Figure S12. Presence of apatite is still apparent in both kidney stone spectra. In the initial survey run, $CaCO_3$ appears to be amorphous or mixed calcite/amorphous as peaks are shifted to lower energy and broadened compared to pure calcite.[1] After polishing and/or time, the spectrum matches that of calcite. Calcite can be distinguished from aragonite by the presence of the peak at 280 cm^{-1}.

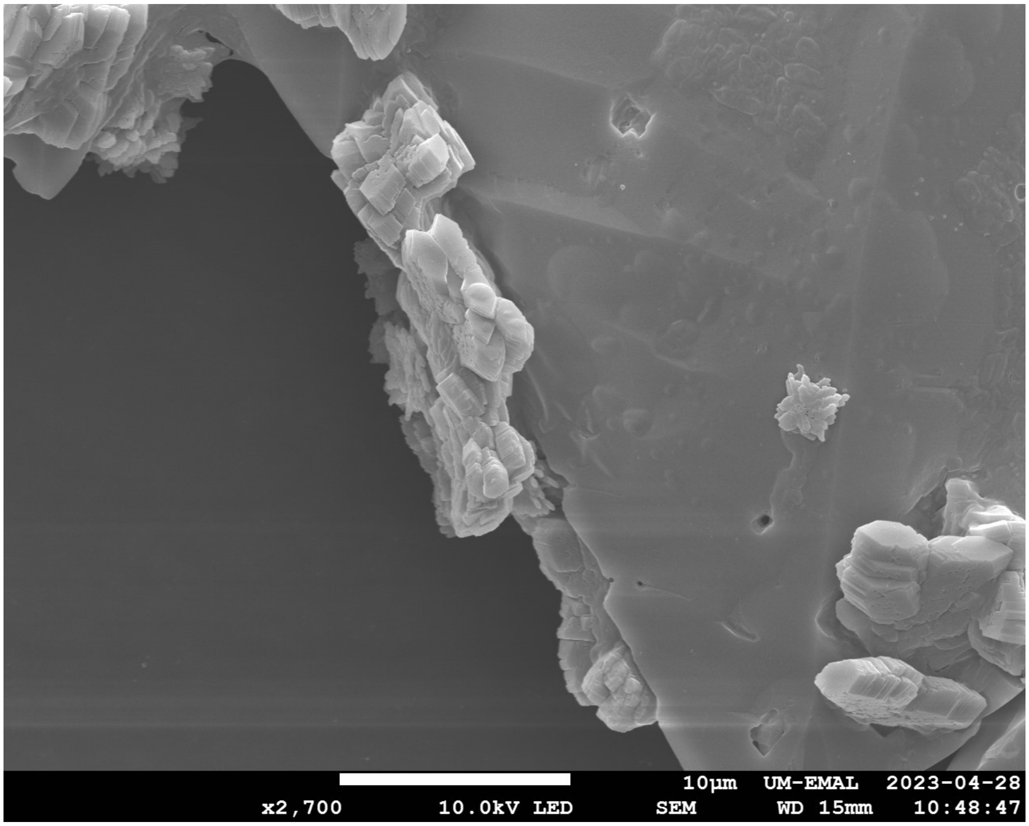

Figure S14: Scanning electron microscopy image of stacked hexagonal plates of calcium oxalate monohydrate growing on a calcium oxalate dihydrate blade.

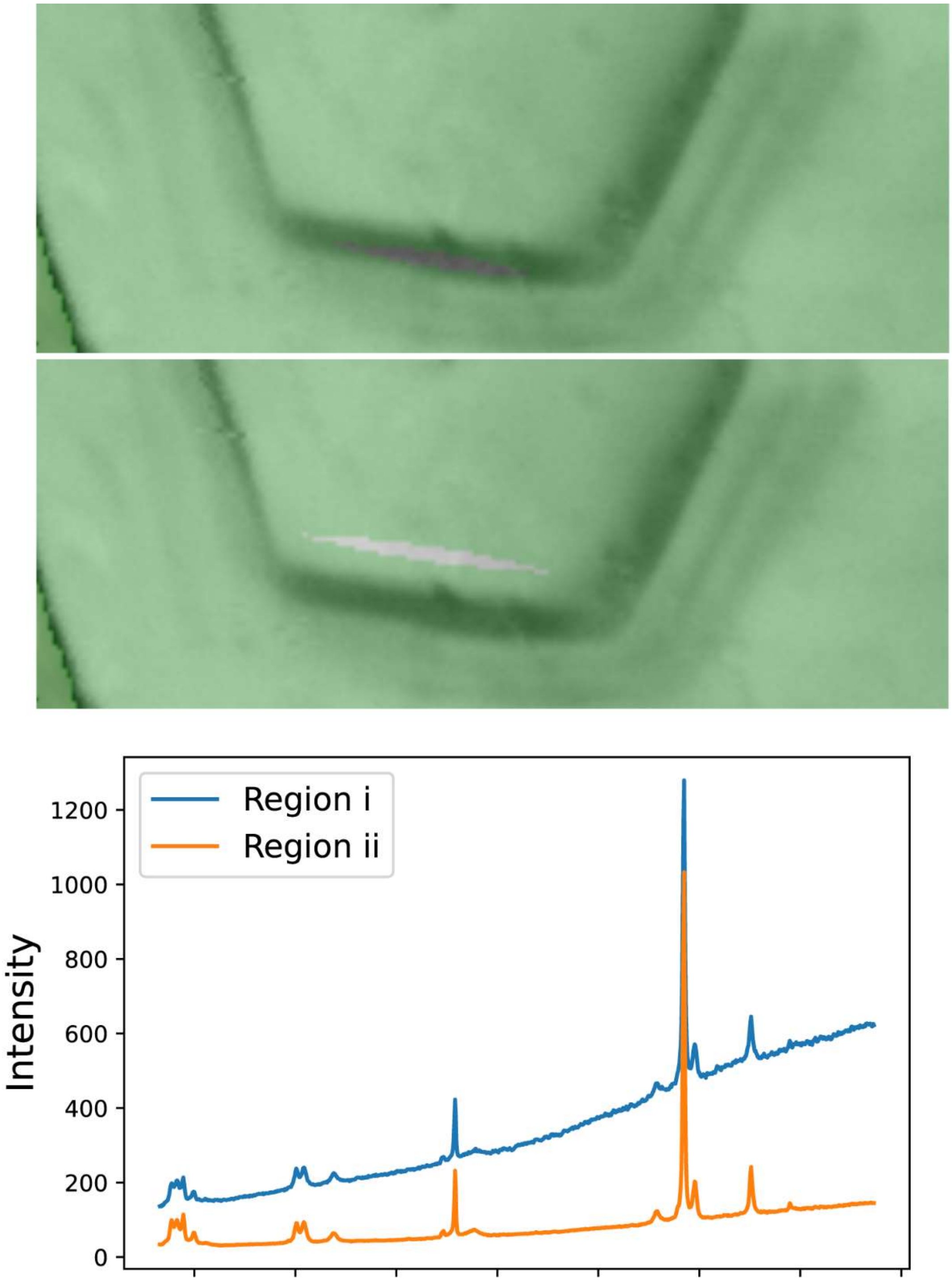

Figure S15: COM spectra of unmasked regions (grey areas) in COM crystal in Figure 5, Region C. Autofluorescence is higher in darker regions of this crystal, potentially indicating increased concentration of organic material.

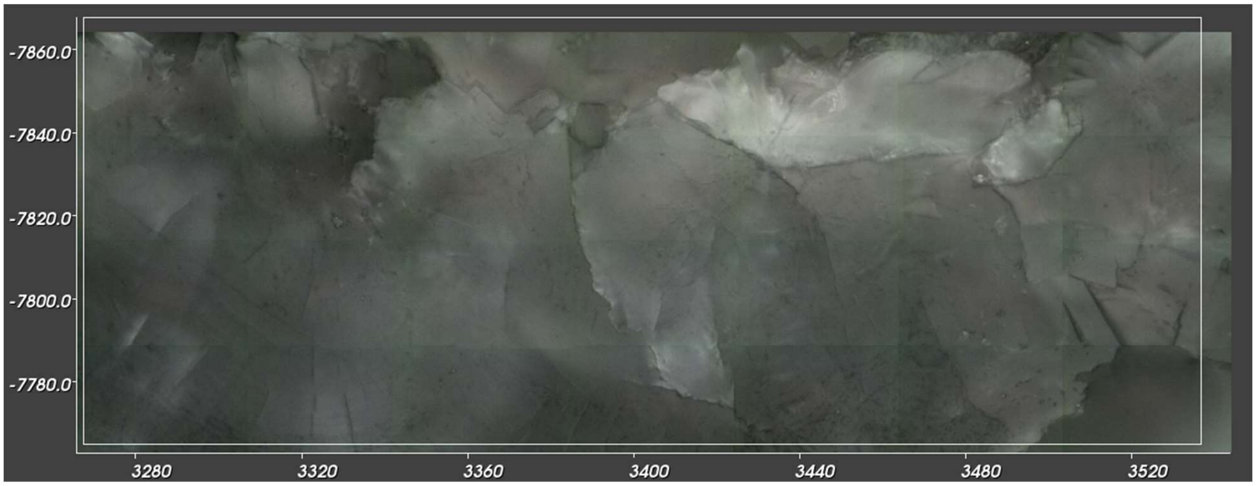

Figure S16: White light image of sample in Figure 6.

Additional References

(1) Weiner, S.; Levi-Kalisman, Y.; Raz, S.; Addadi, L. Biologically Formed Amorphous Calcium Carbonate. *Connect. Tissue Res.* **2003**, *44* (1), 214–218. https://doi.org/10.1080/03008200390181681.
